# The efficacy of PI3Kγ and EGFR inhibitors on the suppression of the characteristics of cancer stem cells

**DOI:** 10.1038/s41598-021-04265-w

**Published:** 2022-01-10

**Authors:** Yanning Xu, Said M. Afify, Juan Du, Bingbing Liu, Ghmkin Hassan, Qing Wang, Hanbo Li, Yixin Liu, Xiaoying Fu, Zhengmao Zhu, Ling Chen, Masaharu Seno

**Affiliations:** 1 Department of Pathology, Tianjin Central Hospital of Gynecology Obstetrics, Nankai University Affiliated Maternity Hospital, Tianjin Key Laboratory of Human Development and Reproductive Regulation, Tianjin, 300100 China; 2 Department of Medical Bioengineering, Graduate School of Natural Science and Technology, Okayama University, Okayama, 700-8530 Japan; 3 Laboratory of Nano-Biotechnology, Graduate School of Interdisciplinary Science and Engineering in Health Systems, Okayama University, Okayama, 700-8530 Japan; 4 Division of Biochemistry, Chemistry Department, Faculty of Science, Menoufia University, Shebin El Koum-Menoufia, 32511 Egypt; 5 Department of Etiology, Shanxi Provincial Cancer Hospital, Taiyuan, 030013 China; 6 Department of Genetics and Cell Biology, College of Life Sciences, Nankai University, Tianjin, 300071 China; 7 Okayama University Research Laboratory of Stem Cell Engineering in Detroit, IBio, Wayne State University, Detroit, MI 48202 USA; 8 Laboratory of Natural Food & Medicine, Co., Ltd, Okayama University Incubator, Okayama, 700- 8530 Japan

**Keywords:** Cancer, Molecular medicine

## Abstract

Cancer stem cells (CSCs) are capable of continuous proliferation, self-renewal and are proposed to play significant roles in oncogenesis, tumor growth, metastasis and cancer recurrence. We have established a model of CSCs that was originally developed from mouse induced pluripotent stem cells (miPSCs) by proposing miPSCs to the conditioned medium (CM) of cancer derived cells, which is a mimic of carcinoma microenvironment. Further research found that not only PI3K-Akt but also EGFR signaling pathway was activated during converting miPSCs into CSCs. In this study, we tried to observe both of PI3Kγ inhibitor Eganelisib and EGFR inhibitor Gefitinib antitumor effects on the models of CSCs derived from miPSCs (miPS-CSC) in vitro and in vivo. As the results, targeting these two pathways exhibited significant inhibition of cell proliferation, self-renewal, migration and invasion abilities in vitro. Both Eganelisib and Gefitinib showed antitumor effects in vivo while Eganelisib displayed more significant therapeutic efficacy and less side effects than Gefitinib on all miPS-CSC models. Thus, these data suggest that the inhibitiors of PI3K and EGFR, especially PI3Kγ, might be a promising therapeutic strategy against CSCs defeating cancer in the near future.

## Introduction

CSCs have the multilineage differentiation potential and the ability of self-renewal. Since the CSCs can differentiate and generate heterogeneous cell populations to constitute the tumor, they are considered as the tumor-initiating cells^1^. Increasing evidences reveal that CSCs in tumors are responsible for the resistance to chemotherapy and for the tumor relapse^2,3^. Therefore, CSC targeting should be more feasible strategy to treat cancers.

Based on the hypothesis of cancer-inducing^4^, our lab established CSC models from miPSCs through epigenetic regulations affected by the conditioned medium (CM) prepared from various cancer derived cells without any genetic manipulations. The CSC model developed by the treatment with the CM of Lewis lung carcinoma (LLC) cells for 4 weeks was named miPS-LLCcm^5,6^. Then we produced pancreatic CSC model by the exposure to CM of human pancreatic carcinoma cell lines PK8 cells named miPS-PK8cm^7^. We also got breast CSC model by the exposure to CM of Luminal A type human breast cancer cell line T47D cells named miPS-T47Dcm^8^. The converted CSC models exhibited the self-renewal, differentiation potential of some specific phenotypes and potential to form malignant tumors with metastasis. Furthermore, CSC models sustained undifferentiated state expressing genes associated with the stemness such as *Nanog, Sox2, Oct4, Klf-4* and *c-Myc*^5^. Simultaneously, the CSC markers such as *CD24a, EPCAM*, and *CD133* were found significantly elevated in miPS-CSC models but not in miPSCs.

The epigenetic changes play a critical role in the development of CSCs as tumor initiation^9^, which were likely to be mimicked by the conversion of miPSCs into CSCs. Our group demonstrated that DNA hypomethylation was the main cause in the conversion of miPSCs into CSCs. Further bioinformatics analysis of KEGG pathways identified the genes upregulated in the category of class IB PI3K^10^. We also showed that class IA PI3Ks as the responsible gene in the obtained CSC models^11^. These findings were inconsistent with the reports describing that PI3K genes were critical effectors of carcinogenesis in many cancer types^12,13^. Therefore, the CSC model developed from miPSCs are conceivable to evaluate the anti-CSC strategy targeting PI3K-Akt as an important pathway in CSCs.

To date, scientists have identified various PI3Ks and classified them into class I, II and III according to their cellular regulation, molecular structures and the specificities of substrate in vivo^14,15^. Class I PI3Ks are now the most extensively studied among three classes of PI3Ks and are further divided into class IA and class IB based on the differences of regulatory proteins and upstream signaling pathways. Class IA PI3Ks include three catalytic subunits, p110α, p110β and p110δ, and they bind to the SH2-containing regulatory subunit p85^15–17^. PI3Kγ is a single member of class IB PI3K and is primarily driven by the activation of Gαi-coupled GPCRs. PI3Kγ is involved in numerous intracellular signaling pathways, it has been considered as a promising drug target for the treatment of immune diseases and certain cancers recently. In contrast, class II and class III PI3Ks are less known^18^.

Eganelisib is the first and only one PI3Kγ inhibitor to enter the clinical trial NCT02637531 and is currently in Phase I clinical evaluation for mainly advanced solid tumors. Targeted inhibition of Eganelisib on PI3Kγ can inhibit tumor growth and restore antitumor immune responses^19^. But whether it is effective on CSCs is still uncertain. In this study, we intend to study the anti-tumor effect of Eganelisib on the stem cell level, in the hope of treating cancer more effectively, meanwhile breaking through the chemo-resistance and tumor relapse.

Gefitinib, which is one of the epidermal growth factor receptor (EGFR) small molecule tyrosine kinase inhibitor tyrosine kinase inhibitors (TKIs), is widely used for patients with non-small cell lung cancer (NSCLC) harboring EGFR mutations^20^, including exon 19 deletion and exon 21 L858R mutation. However, numerous patients cannot benefit or continue to benefit from EGFR-TKI monotherapy due to primary and secondary drug resistance after a period of treatment^21^. CSCs were found enriched and the EGFR mutant cells acquired resistance during the treatment with EGFR inhibitors^22^. However, the mechanism of acquiring resistance is complexed and still unclear. The significant inhibitory effect of Gefitinib on CSCs themselves has not yet been uncovered.

In the current study, we attempted to evaluate whether Eganelisib and/or Gefitinib exerted antitumor actions against CSC using our novel models in vitro and in vivo.

## Methods

### Cell lines and cell culture

miPS cell (iPS-MEF-Ng-20D-17; Lot No. 012) were provided by the RIKEN Cell Bank, Japan. All cells were incubated in a humidified atmosphere containing 5% CO_2_ at 37 °C. miPS-CSCs (miPS-LLCcm, miPS-T47Dcm and miPS-PK8cm) models were maintained in miPS cells media (15% FBS-Gibco, Ireland, 2 mM l-Glutamine, 0.1 mM 2-mercaptoethanol, 0.1 mM NEAA, 50 U/ml penicillin and 50 U/ml streptomycin, without LIF) on gelatin-coated dishes (0.1% gelatin solution at 37 °C for 30 min). The miPS cells under the control of the *Nanog* promoter expressed GFP were maintained in miPS cells media with LIF on feeder layers.

### Cell proliferation and viability assays

miPS-CSCs were seeded with a density of 1 × 10^4^ cells/well on 96-well plates coated with gelatin. The concentrations of Gefitinib (Selleck, ZD1839) and Eganelisib (Selleck, S8330) were formulated according to the manufacturer’s instructions. After 24 h, test compounds were added at a concentration gradient of 3.125 μmol/L, 6.25 μmol/L, 12.5 μmol/L, 25 μmol/L and 50 μmol/L. Cell viabilities at 48 h were determined as follows. After incubation, 3-(4,5-di-methylthiazol-2-yl)-2,5-diphenyltetrazolium bromide, yellow tetrazole (MTT, Sigma-Aldrich) were added to the wells with the final concentration of 0.6 mg/mL and the plate was incubated for 4 h. Formed formazan crystals were dissolved in 10% (w/v) SDS with 0.02 N HCl and incubated overnight. Finally, the absorbance of each well was measured at 570 nm using an MTP-800AFC microplate reader (Corona, Japan). Viable cells were evaluated by MTT assay as described above. The half-maximal inhibitory concentration (IC50) values were determined based on the survival curve obtained by MTT assay. The experiment was independently repeated three times.

### Cell apoptosis assay

In vitro cell apoptosis was analyzed by flow cytometry (AccuriTM C6 Plus flow cytometer, BD Biosciences) using a Annexin V-Phycoerythrin (PE) and 7-amino-actinomycin (7-AAD) Apoptosis Detection Kit (BD Biosciences, 559763) after three kinds of miPS-CSCs cells treated with inhibitors for 48 h, we analyzed proportions of apoptotic cells using FlowJo Software (Treestar Inc., San Carlos, CA), whereas in vivo apoptotic tumor cells were detected using a Colorimetric TUNEL Apoptosis Assay Kit (Beyotime, C1091).

### Western blot analysis

The cells treated with Gefitinib and Eganelisib for 48 h were collected and resuspended in cell lysis buffer containing 50 mM Tris (pH 7.4), 150 mM NaCl, 1 mM EDTA, 1 mM EGTA, 1 mM NaF, 1 mM Na_3_VO_4_, 1% Triton X-100, 10% glycerol, 0.25% deoxycholate, and 0.1% SDS. Lysates were electrophoresed using SDS-PAGE and blotted onto nitrocellulose (NC) membrane were blocked with 5% nonfat milk or 5% BSA solution for 2 h. The membranes were cut prior to hybridisation with different antibodies (This was the reason for the absence of images of adequate length). Samples were probed with primary antibodies overnight at 4 °C. Secondary antibodies HRP conjugated donkey anti-Rabbit IgG (GE Healthcare NA934V) or goat anti-mouse IgG (H + L) (ZB2305) were diluted at 1:5000. Blots were photographed by the Image Quant LAS 4000 luminescent image analyzer (General Electric, Fairfield, CT). All Western blots were quantified using the Image J program (NIH, USA)^23^. For all the western blots, a statistical analysis based on at least three different experiments.

### Cell invasion and migration assay

According to the manufacturer's instructions for invasion/migration assays, 4 × 10^4^ cells were suspended in the upper chambers of 24-well Transwell plates with/without pre-coated Matrigel (Corning, New York, NY) respectively. 200 μL serum-free medium were plated into the top chamber. The lower chambers were filled with 600 μL serum-containing (10%) medium in each well. Migrated and invaded cells (after incubation for 24 h and 48 h, respectively) on the bottom side of the chamber membrane were fixed and stained with crystal violet and counted under a microscope.

### Soft agar colony formation assay

The miPS-LLCcm, miPS-T47Dcm, miPS-PK8cm cell were cultured in RPMI-1640 with 10% FBS in 6-well plates within a 0.35% agar layer, and 2 × 10^3^ cells were seeded to the middle layer of the soft agar (Lonza, Rockland, USA)^23^. The plates were incubated for 14 d, after which the cultures were inspected and photographed. Assays were conducted in triplicate in a single experiment, and then as three independent experiments.

### In vivo study

All animal studies were approved by the Institutional Animal Care and Use Committee at Nankai University. All the study was carried out in compliance with the ARRIVE guidelines.

Female 3 to 4-week-old BALB/c nu/nu athymic mice (Vitalriver Beijing, China). BALB/c nude mice were transplanted subcutaneously with 1 × 10^6^ miPS-LLCcm cells. When the tumor volume reached approximately 100 mm^3^, 0.1% Tween 80, Gefitinib (100 mg/kg, QOD), Eganelisib (5 mg/kg, QOD), or Gefitinib and Eganelisib (n = 7 mice per group) were administrated and observed for 12 d. The mice were euthanized by isoflurane-euthanasia method (Laboratory Animal Anaesthesia (3rd Ed.) Paul A. Flecknell 2009). 5% of isoflurane was exposed to mice and the exposure was continued until one minute after their breathing stopped. Finally, euthanasia was confirmed by cervical dislocation. The tumor tissues and organs (heart, spleen, lung, liver and kidney) were dissected and weighed. Then the tumors were fixed with formalin and embedded in paraffin. The paraffin blocks were sectioned into 5 μm-thick slices and stained with 0.5% Hematoxylin (Sigma Aldrich) and Eosin Y (Sigma Aldrich).

### IHC in xenograft tumors

Tissue sections of xenograft tumor (4 μm thick) were immunostained with anti-Ki-67 (1:500) antibodies overnight at 4 °C. The peroxidase conjugated streptavidin complex method was performed, followed by the 3, 3′ diaminobenzidine (DAB) procedure according to the manufacturer’s protocols (Dako, Agilent pathology solutions)^23^.

### Statistical analysis

All in vitro experiments were repeated at least three times unless stated otherwise. All values are given as mean ± SD of not less than three measurements (unless otherwise stated). Statistical were drawn using Tukey’s multiple comparisons test and performed using SPSS 21. All statistical tests were two-sided, and differences were considered statistically significant at P < 0.05 unless stated otherwise.

## Results

### Several signaling pathways were activated in miPS-CSCs mode

**Figure 1:**
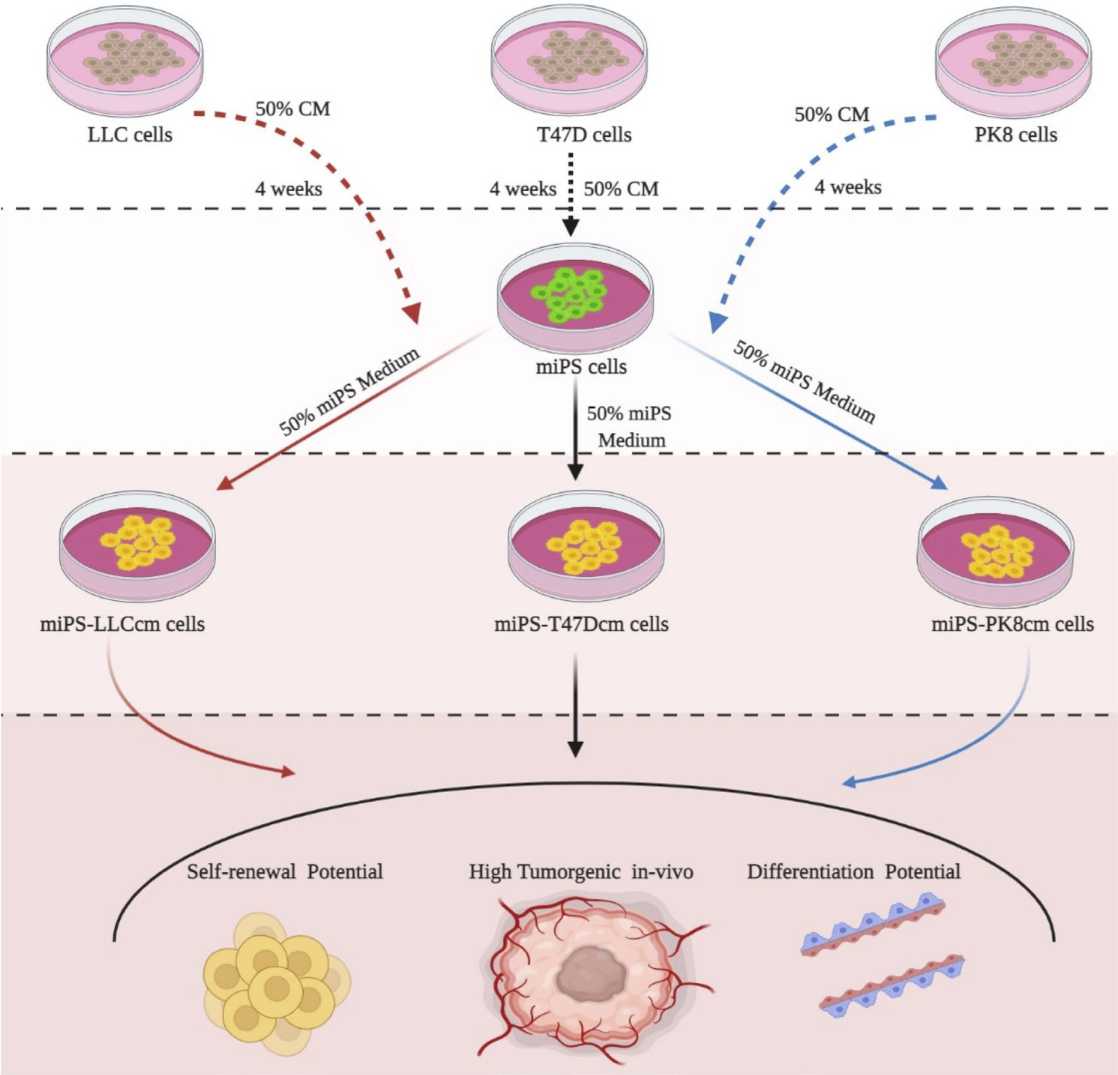
Schematic overview of the preparation of CSCs subjected to this study.

Previously, our group developed miPS-LLCcm cells^5^, miPS-T47Dcm^8^ and miPS-PK8cm cells^7^ as CSCs from miPSCs exposed to the CM of LLC, T47D cells and PK8 cells respectively (Fig. 1). These cells were evaluated for their potential of self-renewal and differentiation together with the stemness markers and confirmed as the CSC models. Allografts of these converted cells developed malignant tumors.

**Figure 2:**
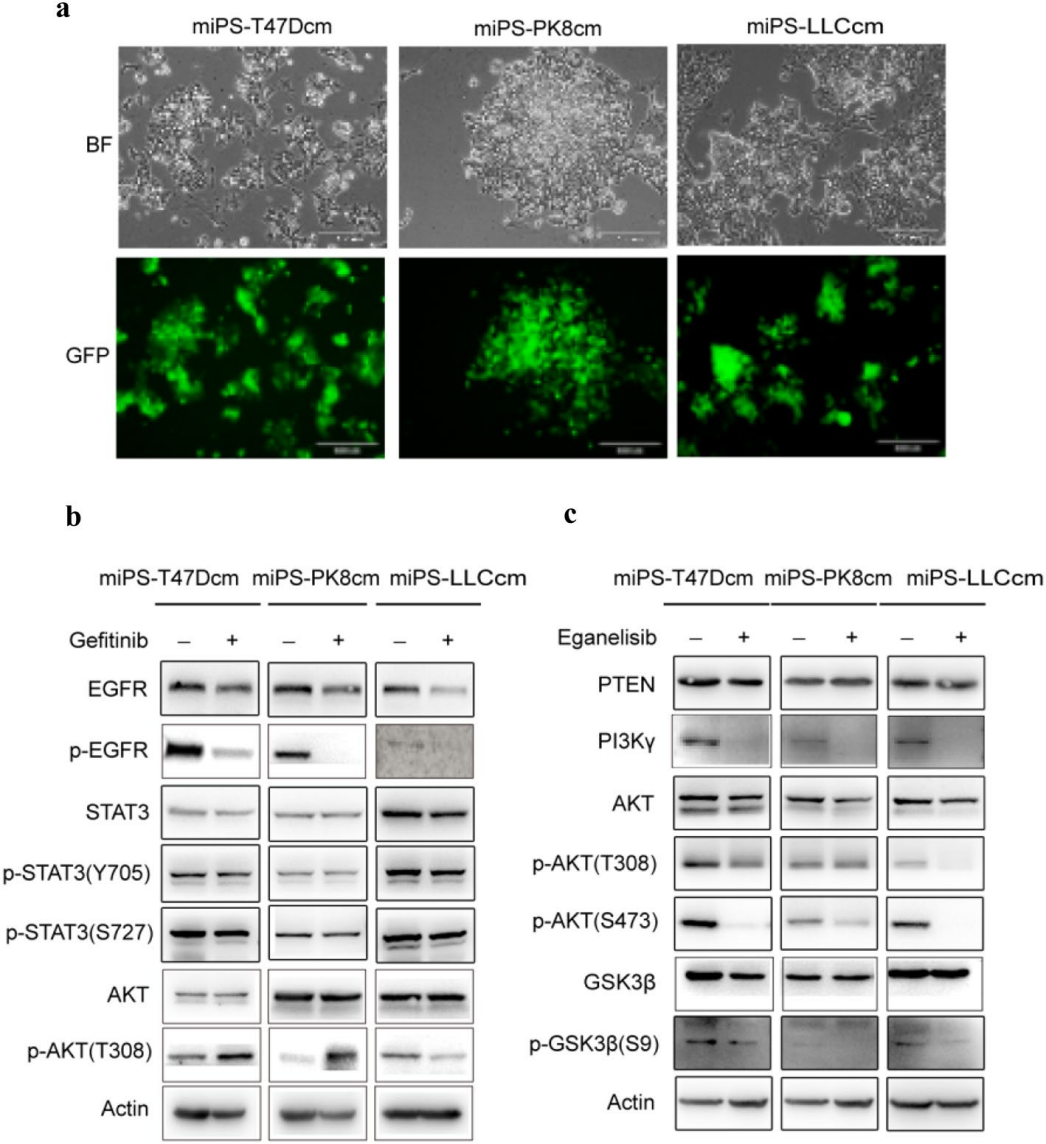
The expression of EGFR and PI3K/AKT pathway related proteins in CSCs derived from iPSCs. (**a**) Representative images for adherent culture of miPS-T47Dcm cells, miPS-LLCcm cells and miPS-PK8cm cells. Scale bars represent 100 μm. **(b)** Western blot analysis of EGFR and partial PI3K/Akt signaling pathway-related protein expressions in the three kinds of miPS-CSC models after treated with Gefitinib (The concentration we used was11.0 μM in miPS-LLCcm, 6.5 μM in miPS-PK8cm, 34.7 μM in miPS-T47Dcm for 48 h). **(c)** Western blot analysis of PI3K/Akt signaling pathway-related protein expressions in the three kinds of miPS-CSC models after treated with Eganelisib (The concentration we used was 14.6 μM in miPS-LLCcm, 18.1 μM in miPS-PK8cm, 16.38 μM in miPS-T47Dcm for 48 h). Gels were cropped. Original western blot figures were presented in Supplementary Figs. S4–S5 online. The band density have been quantified in Supplement Figs. S6 and S7 online. The western blots based on at least three different experiments.

These three CSC models exhibited two different subpopulations of GFP-expressing colonies surrounded by the progenies of myofibroblast-like cells (Fig. 2a). These subpopulations were considered to be to be responsible for the heterogeneity in tumors. In our previous studies PI3 kinasegenes were found overexpressed constitutively activating the PI3K-Akt-mTOR signaling pathway in the CSC models^10,12^.

In the current study, we further assessed changes in the signaling pathways and found that the expression of EGFR, total STAT3, p-STAT3 (on Y705 and S727 site), which were important factors in EGFR signaling pathway^24,25^, were all obviously up-regulated in CSC (Supplementary Fig. S2, gels were cropped, original western blot figures were presented in Supplementary Fig. S3 online).

### PI3K-Akt-mTOR and EGFR signaling pathways were inhibited by inhibitors in miPS-CSC model

We treated all three types of miPS**-**CSC models by inhibitors in vitro. After the treatment with Gefitinib, we found that the expression of EGFR and p-EGFR was obviously down-regulated in all three types of miPS**-**CSC models. In addition, the total STAT3, p-STAT3 (Y705 and S727 site) were slightly inhibited in three kinds of miPS**-**CSCs model (Fig. 2b, gels were cropped, original western blot figures were presented in Supplementary Fig. S4 online). We also observed that the Gefitinib not only significantly inhibited the expression of EGFR in all three types of CSC models, but also activated the phosphorylation of AKT at T308 in PI3K/Akt signaling pathway in miPS-T47Dcm and miPS-PK8cm cells while it was suppressed in miPS-LLCcm cells. The total AKT was no significant changes in all three kinds of miPS-CSC models. After treatment with Eganelisib, we found that the expression of PI3Kγ were obviously down-regulated together with p-AKT (on S308 and S473 site), p-GSK3β (S9) in all three types of miPS**-**CSC models. The total AKT was slightly inhibited and there were no significant changes in PTEN and GSK3β in all three kinds of miPS-CSC models (Fig. 2c, gels were cropped, original western blot figures were presented in Supplementary Fig. S5 online).

### Growth inhibition of miPS-CSC model by Gefitinib and Eganelisib

**Figure 3:**
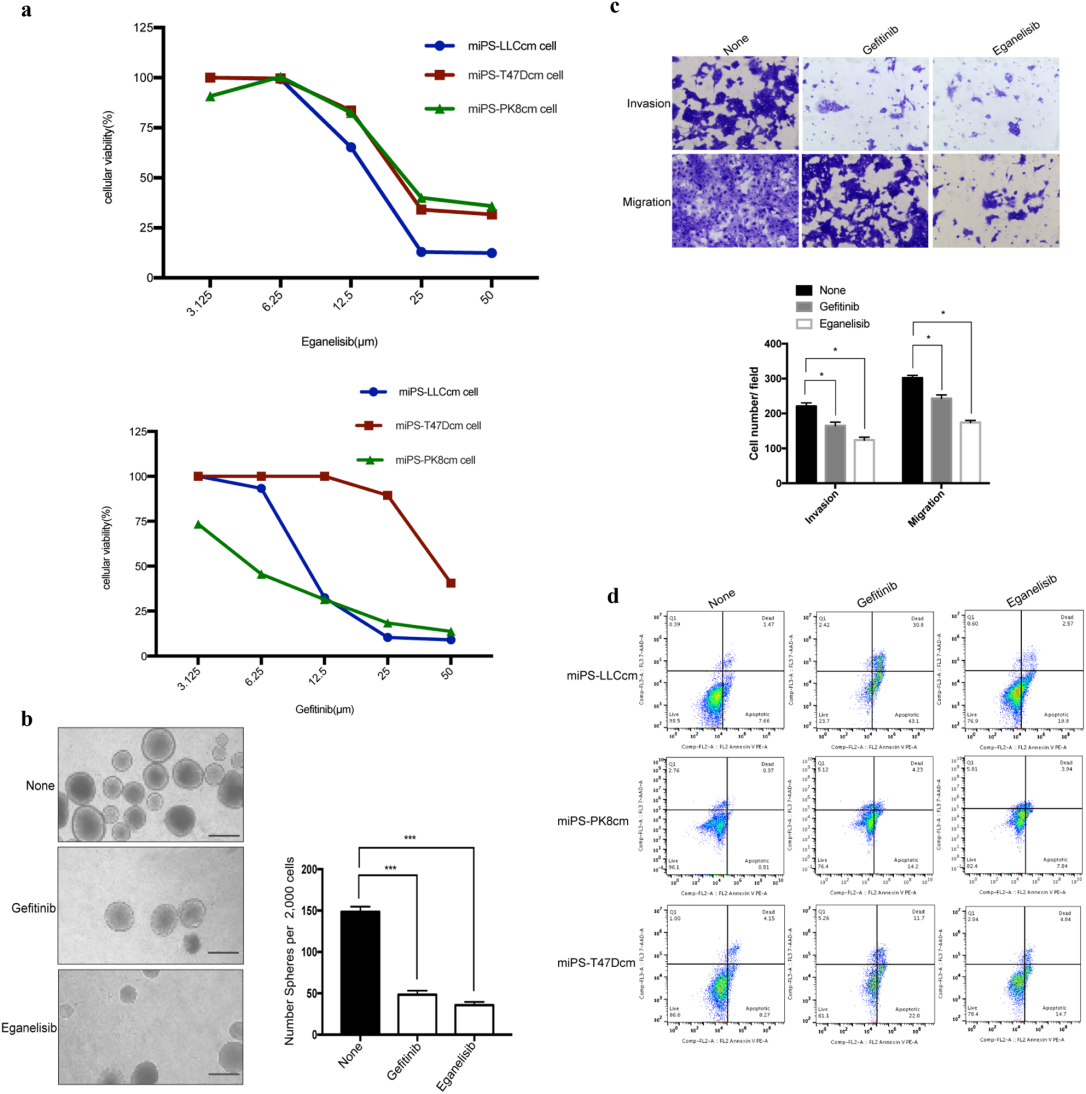
The effects of inhibitors on proliferation, apoptosis, stemness, invasion and metastasis of miPS-CSC models in vitro. **(a)** The proliferation of miPS-CSC models after treated with Eganelisib and Gefitinib was measured by MTT assay. **(b)** The number of spheres with a diameter greater than 75 μm after miPS-CSC models treated with Eganelisib and Gefitinib for 48 h. The data were presented as the number of defined mammospheres per 2000 seeded cells. Representative bright field images for spheres were shown for each group. Scale bar: 100 μm. **(**c**)** miPS-CSC models were subjected to migration and invasion assays after treated with Eganelisib and Gefitinib, 100X. Mean ± s.d. for three independent replicates. **(d)** The apoptosis was analyzed by flow cytometry assay after treated with Eganelisib and Gefitinib. The concentration of Gefitinib we used was 11.0 μM in miPS-LLCcm, 6.5 μM in miPS-PK8cm, 34.7 μM in miPS-T47Dcm for 48 h. The concentration of Eganelisib we used was 14.6 μM in miPS-LLCcm, 18.1 μM in miPS-PK8cm, 16.38 μM in miPS-T47Dcm for 48 h for (**b**), (**c**) and (**d**). The cell was miPS-LLCcm in (**b**) and (**c**). *P < 0.05, ***P < 0.001, Tukey’s multiple comparisons test for (**b**) and (**c**).

miPS**-**CSC models were assessed for the sensitivity to Gefitinib and Eganelisib. As a result, these compounds suppressed the the proliferation ability and the number of the surviving cells were decreased gradually with the increasing concentration gradient of (Fig. 3a). The IC_50_ of Eganelisib was 14.6 μM in miPS-LLCcm, 18.1 μM in miPS-PK8cm, 16.38 μM in miPS-T47Dcm. IC_50_ of Gefitinib was 11.0 μM in miPS-LLCcm, 6.5 μM in miPS-PK8cm, 34.7 μM in miPS-T47Dcm (Supplementary Table S1 online). The IC_50_ of Gefitinib to miPS-T47Dcm was about sixfold higher than the miPS-PK8cm, threefold higher than the miPS-LLCcm. These results indicated Gefitinib was more effective on miPS-PK8cm cells and miPS-LLCcm cells than on miPS-T47Dcm cells whereas miPS-PK8cm cells were the most sensitive to Gefitinib. The sensitivity of all types of miPS**-**CSC models to Eganelisib were quite similar with that to Gefinitib. We also compared the morphological change of miPS**-**CSC models with or without inhibitors. For example, we found miPS-LLCcm cells turned round, lusterless and fluorescence also disappeared according to the increasing concentration of inhibitors (Supplementary Fig. S1 online). In our previous experiments, GFP expression was controlled under *Nanog* promoter to stably express only when the cells were undifferentiated and to be silenced in differentiated or dead cells^26^. These results indicated that Gefinitib and Eganelisib inhibited the proliferation of miPS**-**CSC models in a dose dependent manner.

### Self-renewal of miPS-CSC models was weakened by Gefitinib and Eganelisib

Since the inhibitors was found to reduce the proliferation ability, the effects of Gefitinib and Eganelisib on the potential of self-renewal and/or tumorigenesis of miPS-CSC models were further assessed. miPS**-**CSC models treated with the inhibitors exhibited significant reduction of the sphere numbers with a diameter greater than 75 µm when compared with the no inhibitor group (Fig. 3b). As the results, Gefitinib and Eganelisib could significantly inhibited the self-renewal potential of miPS**-**CSCs model.

### Invasion and migration capacities of miPS-CSC models were weakened by Gefitinib and Eganelisib

In the case of migration, Eganelisib showed higher suppression effect compared to Gefitinib. Cell invasion through the matrigel basement membrane in the cell invasion assay and cell migration directly through the membrane in the cell migration assay were assessed in transwell plate. Cells that invaded to the lower surface of the membrane in these assays were fixed and stained (Fig. 3c). As a result, Gefitinib and Eganelisib significantly decreased the migration and invasion capacity in miPS-CSC models.

### Apoptosis levels of miPS-CSC models were significantly increased by Gefitinib and Eganelisib

The proportions of apoptotic cells were estimated by flow cytometer by the treatment of three kinds of miPS-CSC models with the inhibitors at respective IC_50_s. Both of Gefitinib and Eganelisib exhibited the significant increases of apoptotic cells in miPS-LLCcm cells by 43% with Gefitinib and by 20% with Eganelisib, in miPS-T47Dcm cells by 22% with Gefitinib and by 15% with Eganelisib, in miPS-PK8cm cells by 14% with Gefitinib and 8% with Eganelisib while the cells without treatment exhibited 8%, 1% and 8% in miPS-LLCcm, miPS-PK8cm and miPS-T47Dcm cells respectively (Fig. 3d).

### Gefitinib and Eganelisib inhibited the tumorgenicity in vivo

**Figure 4:**
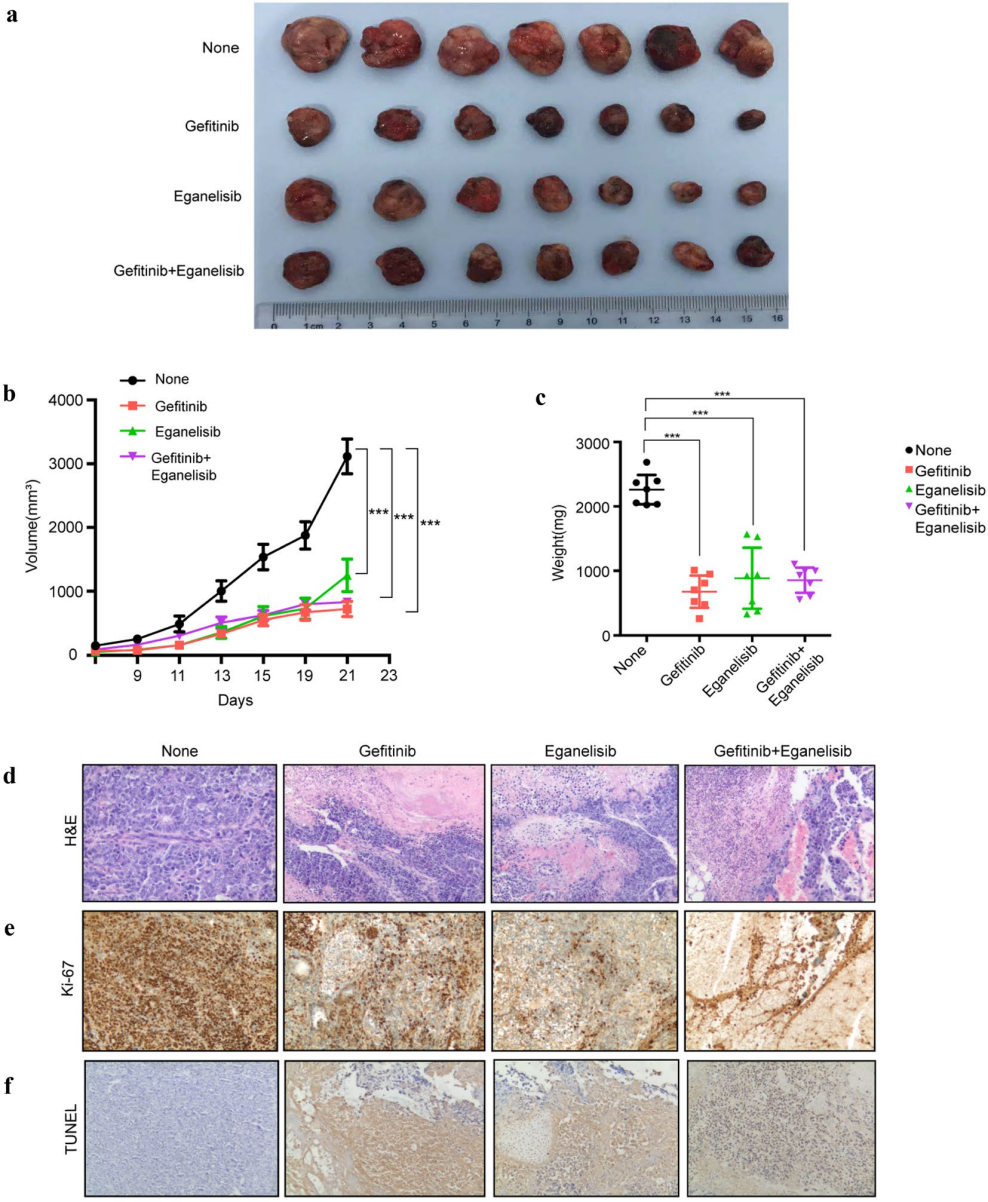
Antitumor effect of inhibitors on miPS-CSC xenografts. **(a)** Isolated xenograft tumors from different groups of mice after treated with inhibitors. BALB/c nude mice were challenged subcutaneously with 1 × 10^6^ miPS-LLCcm cells for 9 days, followed by administration 0.1% Tween 80, Gefitinib (100 mg/kg, QOD), Eganelisib (5 mg/kg, QOD), or combination of Gefitinib plus Eganelisib (7 mice per group) for another 12 days. Ruler scale is in cm. **(b)** Measurement of tumor volume and **(c)** weight from different groups of mice after treated with inhibitors. **(d)** Representative H&E staining of developed tumors treated with or without inhibitors. **(e)** Immunostaining against Ki-67 in tumors from mice treated with or without inhibitors. **(f)** TUNEL assay of tumor from mice treated with or without inhibitors. Original magnification for H&E, Ki-67 and TUNEL staining is × 100. (**b**,**c**) Data were statistically analyzed by Tukey’s multiple comparisons test and expressed as mean ± s.d., where ***P < 0.001.

Since Eganelisib and Gefitinib were found the most effective on miPS-LLCcm cells among the three CSC models, we evaluated the antitumor effects of Eganelisib and Gefitinib on miPS-LLCcm cells in vivo. Injecting miPS-LLCcm cells, tumor xenografts in s.c. were established with athymic nude mice. The mice were randomized to receive Eganelisib and/or Gefitinib when tumors reached an approximate volume of 100 mm^3^. The growth of tumor xenografts were not only suppressed by Eganelisib or Gefitinib but by the combination of both (Fig. 4a). As the result, both the volume and the weight of tumors were significantly decreased in three groups of treatments with P < 0.001 (Fig. 4b,c). All the tumors 12 days after the treatments with the inhibitors exhibited extensive necrosis (Fig. 4d), decreased proliferation, which was confirmed by immunostaining with ki-67 (Fig. 4e) and increased apoptosis, which was assessed by TUNEL assay (Fig. 4f). The apoptosis induced by either Gefitinib or Eganelisib appeared remarkable indicating the therapeutic effects much better than that by the combination.

**Figure 5:**
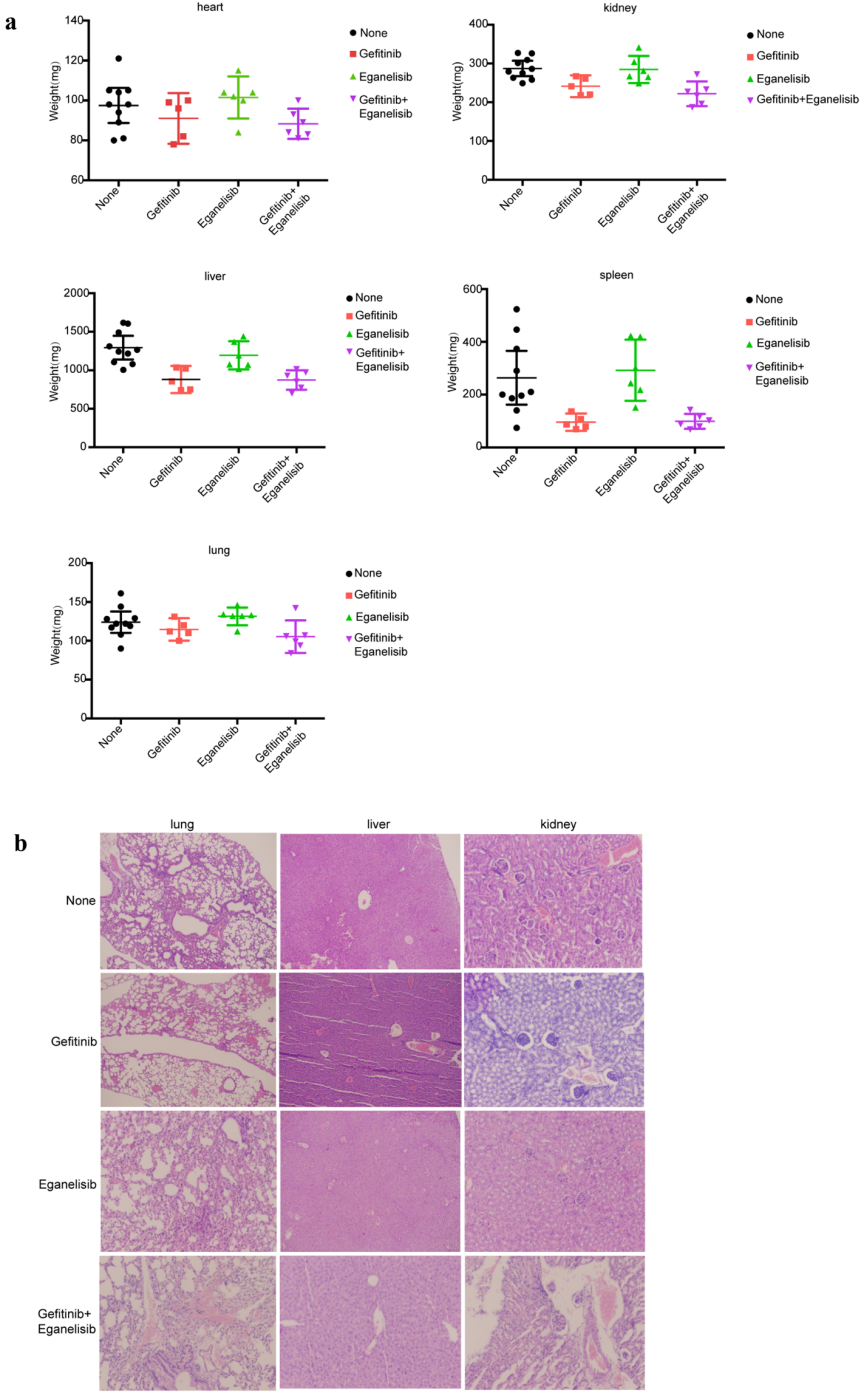
The changes of weight and morphological in important organs from different groups of mice after treated with inhibitors. **(a)** The weight of major organs from mice receiving different treatments of inhibitors. **(b)** Representative H&E picture about morphological changes in the lung, liver and kidney from mice receiving different treatments of inhibitors. Original magnification for H&E is × 100.

As for the side-effects, the mice in the groups treated with Eganelisib showed no obvious weight loss in the major organs when compared to those without treatment (Fig. 5a). No obvious pathological change in these organs treated with Eganelisib as compared to untreated groups (Fig. 5b). On the other hand, treatment with Gefitinib and the combination of Eganelisib with Gefitinib exhibited weight of major organs relatively lower than that of untreated groups as well as hyperemia and hemorrhage were observed in the lung, liver and kindey. The periglomerular space widened was further found in Gefitinib treatment group. Collectively, Eganelisib had less side effects than Gefitinib and Eganelisib combined with Gefitinib.

## Discussion

CSCs are considered as the tumor-initiating cells not only in charge of continuous and uncontrollable growth of malignant tumors but also playing important roles in tumor metastasis, recurrence and acquisition of chemo- and radiation-resistance. Therefore, targeting the CSCs should be more effective strategy to treat the cancer than targeting cancer cells, which are generally heterogeneous and most of all are the progenies of CSCs. Until now the definite origin of CSCs is still obscure although CSCs have been hypothesized to be developed from several origins such as inflammatory microenvironment, genomic instability, cell fusion and lateral gene transfer. Recent studies have revealed that the cancer-inducing microenvironment will be the most important key to establish the logic of CSCs developing from normal stem cells^4^. According to this idea, our group had successfully obtained several CSC models from miPS with the CM prepared from various cancer cell lines. The miPS-CSC models exhibited the basic characteristics of CSCs forming spheroids in suspension culture, differenting into heterogeneous phenotypes of cells, generating and constituting malignant tumors, and so on. The CSC models expressed genes associated with the stemness such as *Nanog, Sox2, Oct4*, and *c-Myc*, as well as those of molecular markers of CSC such as *CD44, CD24a, EPCAM* and *CD133*^4,5,7,8^. With these CSC models, we tried to find several effective anti-CSC drugs.

Generally, the activation of EGFR is known to lead to the phosphorylation of PI3K and the subsequent generation of phosphatidylinositol-3,4,5-trisphosphate (PIP3), which in turn triggers the AKT. The activated AKT can promote cell growth and inhibit apoptosis^14,27–30^. There is a particularly intimate relationship between the PI3K-AKT and EGFR pathways, where inhibitors of one pathway will often activate the other^31,32^. In the current study, we chose Eganelisib and Gefitinib as the inhibitors of PI3Kγ and EGFR, respectively, to evaluate their therapeutic effects and crosstalk on our CSC models.

The CSC models obviously exhibited different sensitivity to Gefitinib with different IC_50_s, which was approximately sixfold higher in miPS-T47Dcm cells than in miPS-PK8cm cells, and threefold higher than in miPS-LLCcm cells. Accordingly, Gefitinib could inhibit the growth of miPS-PK8cm cells in the most efficient manner among the three models. This result was inconsistent with that by Yang’s group^33^. They assessed the effect of Gefitinib on CSCs and non-CSCs, which were separated by CD133 antibody from NSCLC cell line PC9 cells expressing the EGFR exon 19 deletion mutation and sensitive to Gefinitib. And PC9-CSCs were obviously resistant to Gefitinib. Simultaneously, Gefitinib was found to inhibit the entire EGFR/PI3K/Akt signaling pathway in the treatment of PC9-non-CSCs. In their experiments Gefitinib failed to suppress the activation of PI3K/Akt pathway in the PC9-CSCs while the treatment significantly inhibited the phosphorylation of EGFR. In contrast, we found that Gefitinib not only significantly inhibited EGFR signaling pathway in all three CSC models, but also activated phosphorylation of AKT at T308 on PI3K/Akt signaling pathway in miPS-T47Dcm and miPS-PK8cm cells while the phosphorylation of AKT at T308 was suppressed in miPS-LLCcm cells. These differences may explain diversity between the CSC models.

Taking the PC9-CSCs as CSCs selected by a biological marker CD133 from a NSCLC cell line into consideration, there appeared some difficulties to assess all the different effects in a single selected CSCs as demonstrated by Yang^33^. Feedback activation PI3K/AKT by the repression of EGFR signaling induced in miPS-T47Dcm cells showed resistance to Gefitinib. Therefore, our models are conceivable and have advantage to evaluate the effects of drugs on CSCs with inconsistent experimental results.

Activation of the PI3K/Akt/mTOR and mitogen-activated protein kinase (MAPK) pathways was studied in an established paclitaxel (PTX)-resistant gastric cancer (GC) cell subline^34^. The dual PI3K/mTOR inhibitor BEZ235 indicated the therapeutic efficacy in PTX-resistant GC cells both in vitro and in vivo. Activated PI3K/Akt pathways have also been documented in other PTX-resistant cancers in prostate and breast^35,36^. In our study, Eganelisib, a small molecular PI3Kγ inhibitor, displayed significant therapeutic efficiency in all models of CSCs including miPS-T47Dcm cells, which exhibited resistance to Gefitinib. This finding could be a promising instruction to overcome induced chemoresistance in cancer treatment because Eganelisib eradicated the resistance to Gefitinib while further investigation is required.

Kaneda et al.^37^ and other researchers^38^ have shown that highly expressed PI3Kγ promotes the migration of tumor cells and reduces the production of inflammatory mediators in myeloid-derived suppressor cells (MDSCs). Eganelisib has shown not only selective inhibition of PI3Kγ in vivo^39^, but also orally administrative with low clearance and good biosafety in animal studies. Since severe side effects have not been reported the clinical trial phase I, Eganelisib was further pushed forward to the phase II (NCT03795610)^40^. These results were consistent with those in our experiments.

From another perspective, selective inhibition of PI3Kγ reshaped tumor immune microenvironment (TIME) by switching macrophages from the immuno-suppressive M2-like phenotype to the pro-inflammatory M1-like state, restoring sensitivity to immune check-point blocking (ICB) and promoting tumor regression via synergistic activity with cytotoxic T cells. Therefore, Eganelisib have great potential in favor of immunotherapy on cancer.

## Conclusions

Since the CSC models appeared to acquire significance in the signaling pathways as the signature of tumor initiation, they sound feasible to be targeted in cancer treatment. In this paper, a novel approach to target CSCs was proposed effectively reducing self-renewal and tumorgenicity. The PI3K/AKT/mTOR pathway would be a featured candidate to be effectively targeted to defeat cancer^9^. Especially PI3Kγ will be the main but not the only factor that scientists take into account designing PI3K-related inhibitors^41^. From the overall point of view considering the interaction of PI3Kγ with other related targets and signaling pathways such as EGFR comprehensively, providing precise therapeutic strategies is our ultimate goal. Our work sheds a light upon the improvement of tumor chemotherapy providing evidences for further investigation on the tumors with acquired resistance. The development of agents, which can target the molecules potentially reducing drug-resistance and tumor recurrence rate, is very important in order to save patients.

## Supplementary Information

Supplementary Information.

## Data Availability

All data generated or analysed during this study are included in this published article and its Supplementary File.
