# Supplementary material for: The efficacy of PI3Kγ and EGFR inhibitors on the suppression of the characteristics of cancer stem cells
Source: Sci Rep. 2022 Jan 10;12:347. doi: 10.1038/s41598-021-04265-w (PMC8748513; doi:10.1038/s41598-021-04265-w)
Supplement: Supplementary file 1 — Supplementary Information. [file 41598_2021_4265_MOESM1_ESM.pdf]

Title

The efficacy of PI3Kγ and EGFR inhibitors on the suppression of the characteristics of cancer stem cells

Authors

Yanning Xu[1,2,3], Said M. Afify[2,4], Juan Du[5], Bingbing Liu[1], Ghmkin Hassan[2,3], Qing Wang[6], Hanbo Li[1], Yixin Liu[1], Xiaoying Fu[2,3], Zhengmao Zhu[6], Ling Chen[1*], Masaharu Seno[2,3,7,8*]

Affiliations

1. Department of Pathology, Tianjin Central Hospital of Gynecology Obstetrics, Nankai University Affiliated Maternity Hospital, Tianjin Key Laboratory of Human Development and Reproductive Regulation, Tianjin 300100, China.
2. Department of Medical Bioengineering, Graduate School of Natural Science and Technology, Okayama University, Okayama 700-8530, Japan.
3. Laboratory of Nano-Biotechnology, Graduate School of Interdisciplinary Science and Engineering in Health Systems, Okayama University, Okayama 700-8530, Japan.
4. Division of Biochemistry, Chemistry department, Faculty of Science, Menoufia University, Shebin El Koum-Menoufia 32511, Egypt.
5.Department of etiology, Shanxi provincial cancer hospital, Taiyuan 030013, China.
6. Department of Genetics and Cell Biology, College of Life Sciences, Nankai University, Tianjin 300071, China.
7. Okayama University Research Laboratory of Stem Cell Engineering in Detroit, IBio, Wayne State University, Detroit, MI 48202, USA
8.Laboratory of Natural Food & Medicine, Co., Ltd, Okayama University Incubator, Okayama 700- 8530, Japan.

Correspondence: mseno@okayama-u.ac.jp; Tel: +81-86-251-8216

chenlingdoctor@126.com; Tel: +86-22-58287541

	IC50 of Gefitinib (μmol/L)	IC50 of Eganelisib (μmol/L)
miPS-T47Dcm	34.68	16.38
miPS-LLCcm	11	14.62
miPS-PK8cm	6.47	18.13

Supplementary Table S1: IC50 of Gefitinib and Eganelisib in mips-CSCS analyzed by MTT

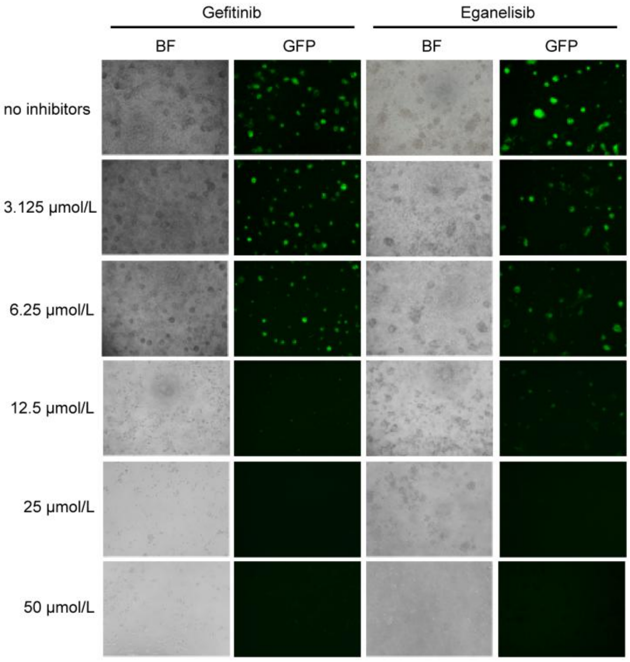

Supplementary Figure S1: Compared the morphological change of miPS-CSC models with or without inhibitors. miPS-LLCcm cells turned round, lusterless and fluorescence also disappeared according to the increasing concentration of inhibitors.

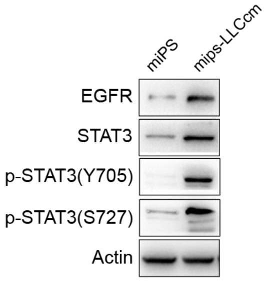

Supplementary Figure S2: Western blot analysis of EGFR signaling pathway-related protein expressions in miPS and miPS-CSCs model (miPS-LLCcm), EGFR mutated in miPS-CSCs cells.

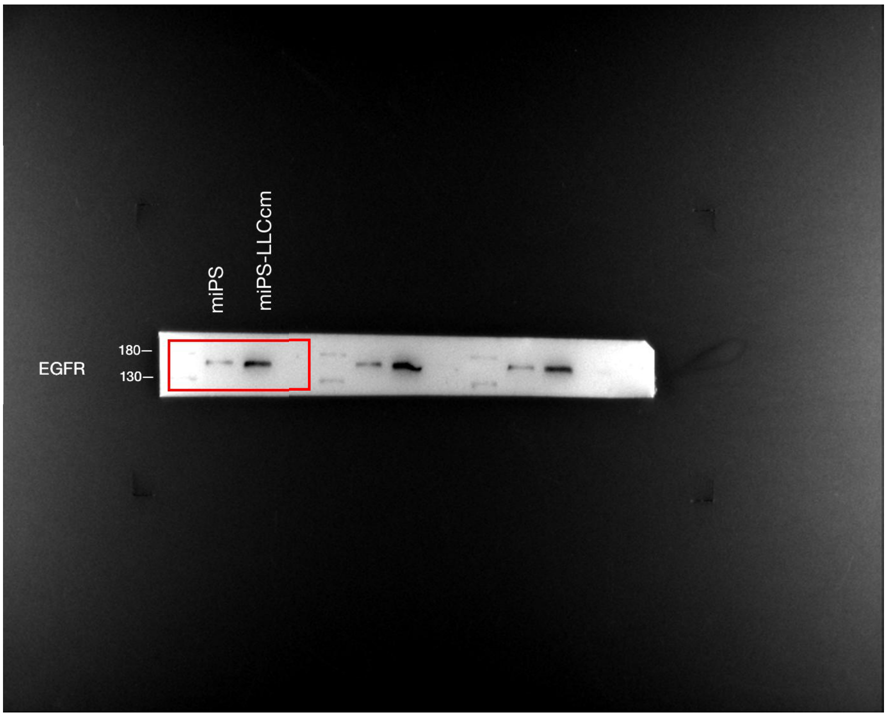

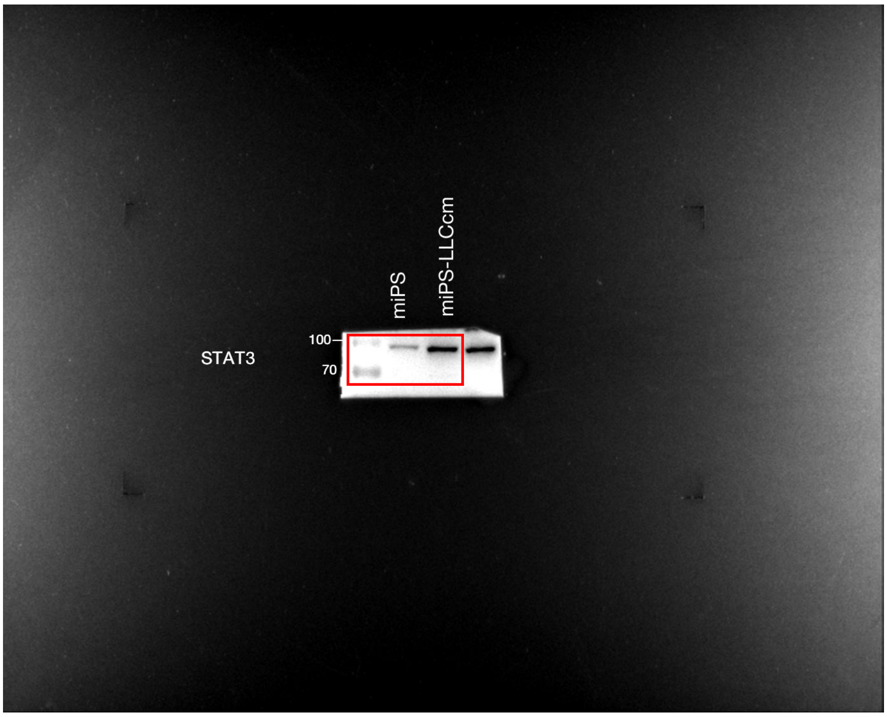
miPS
miPS-LLCcm
STAT3
100
70

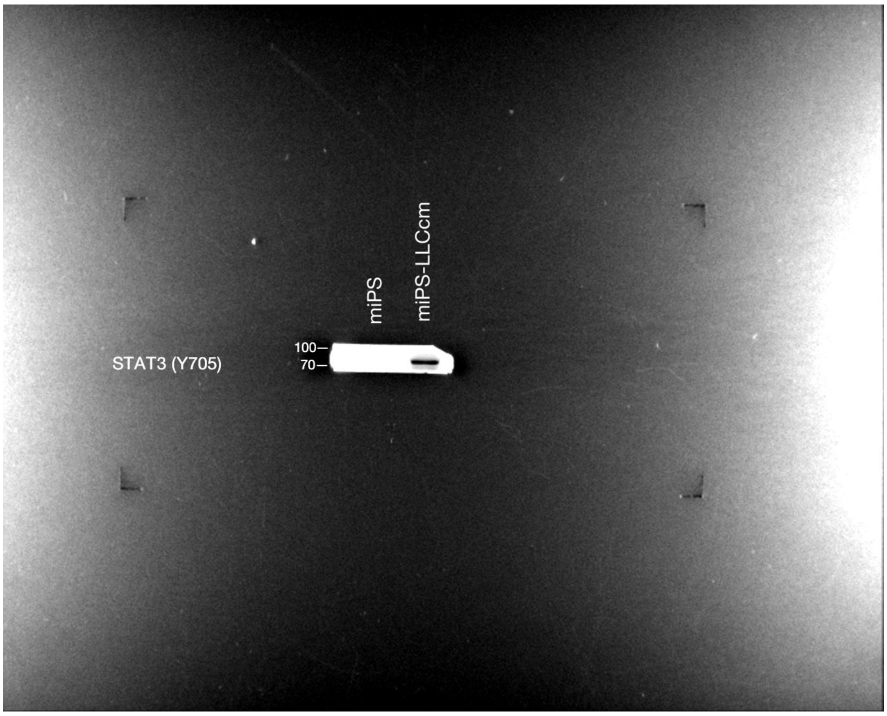
miPS
miPS-LLCcm
STAT3 (Y705)
100
70

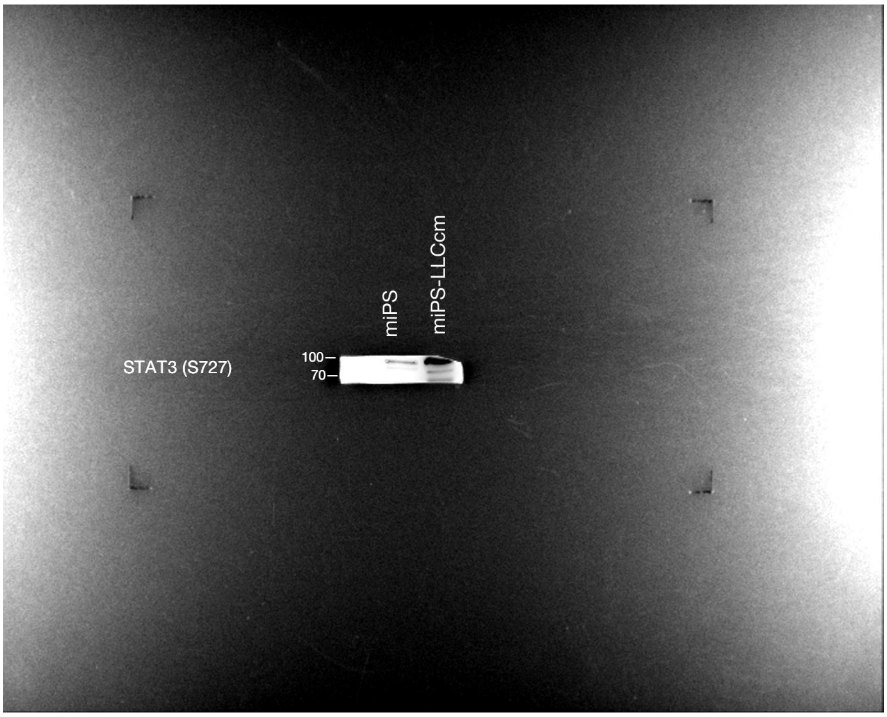

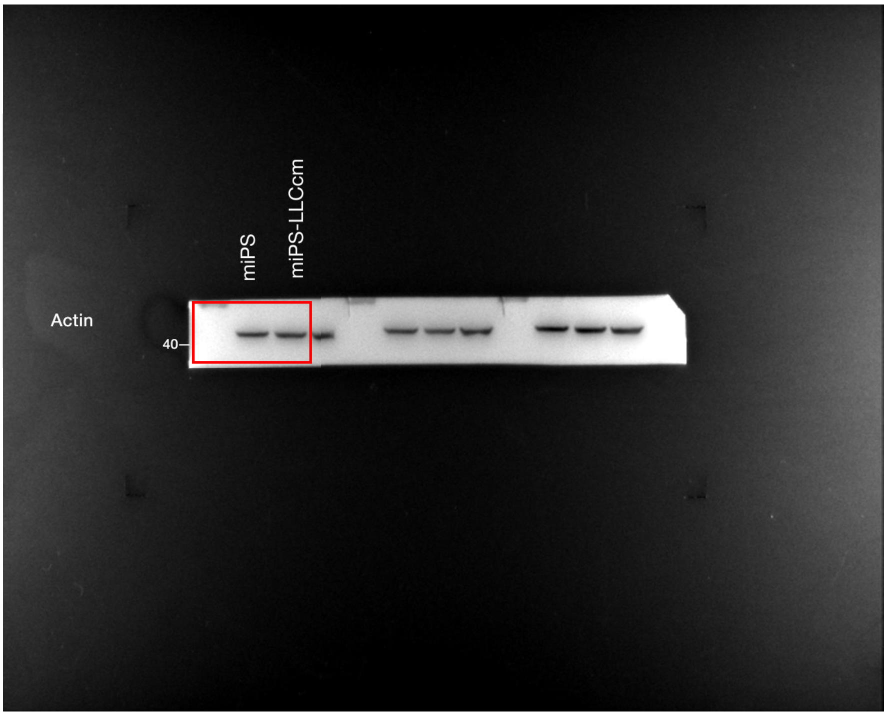

Supplementary Figure S3: Uncropped and unmodified original western blot figures related to **Supplementary Figure S2**.

miPS-T47Dcm

in figure 2b

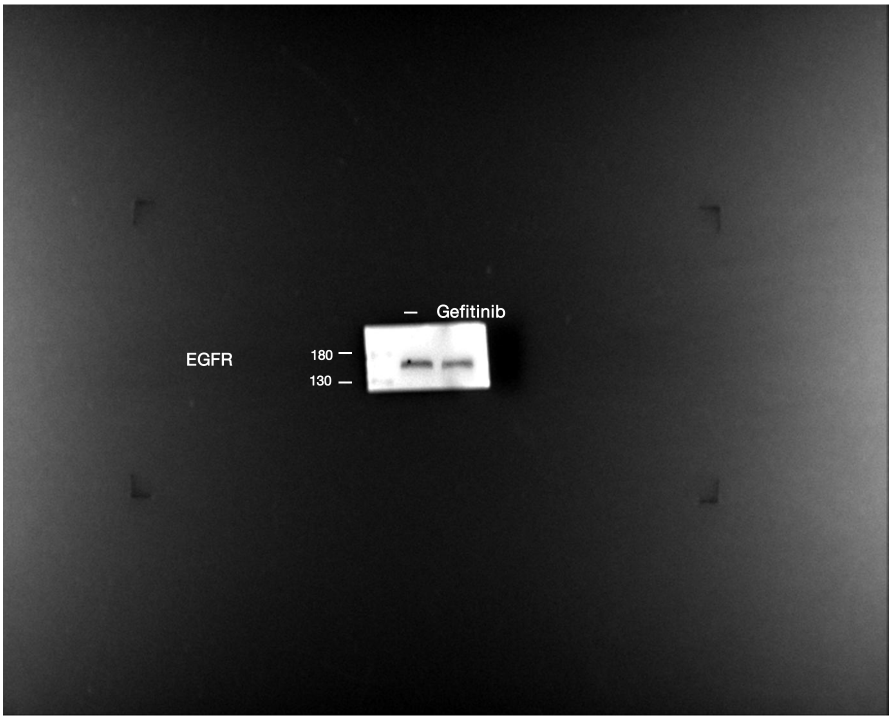

miPS-PK8cm

in figure 2b

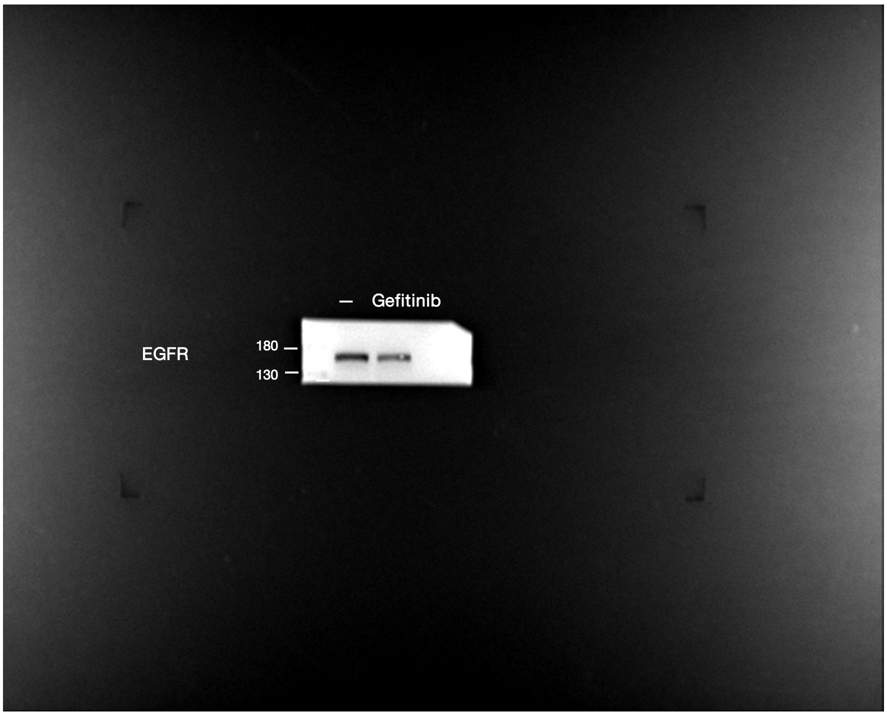

miPS-LLCcm

in figure 2b

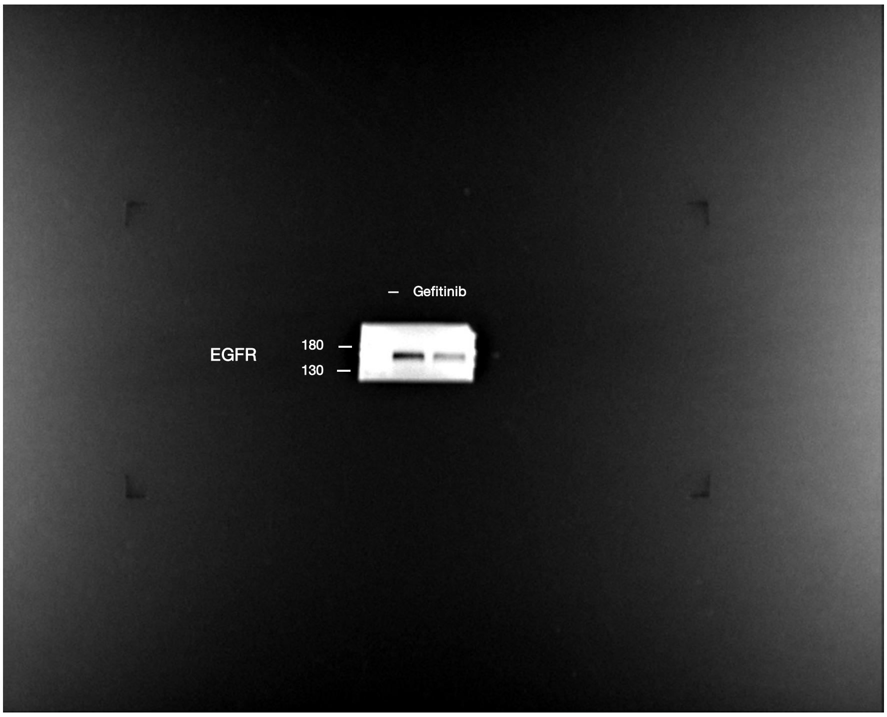

miPS-T47Dcm

in figure 2b

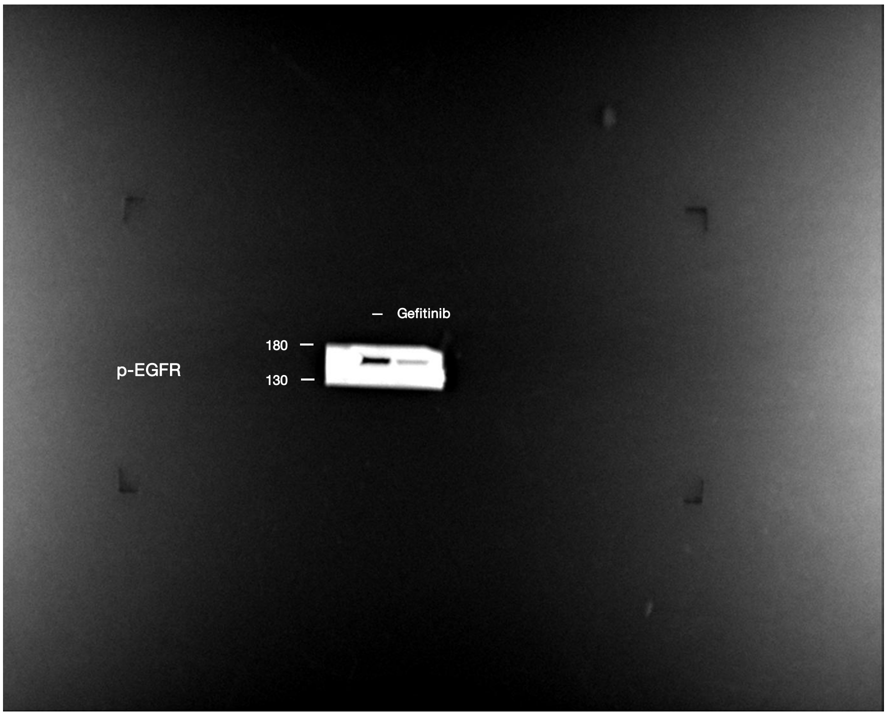

miPS-PK8cm

in figure 2b

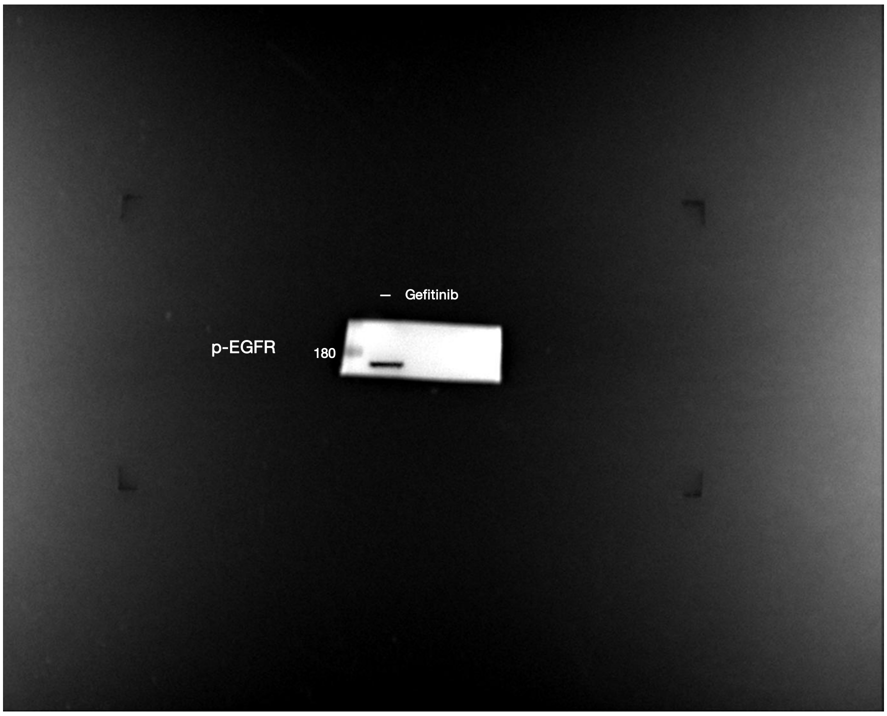

miPS-LLCcm

in figure 2b

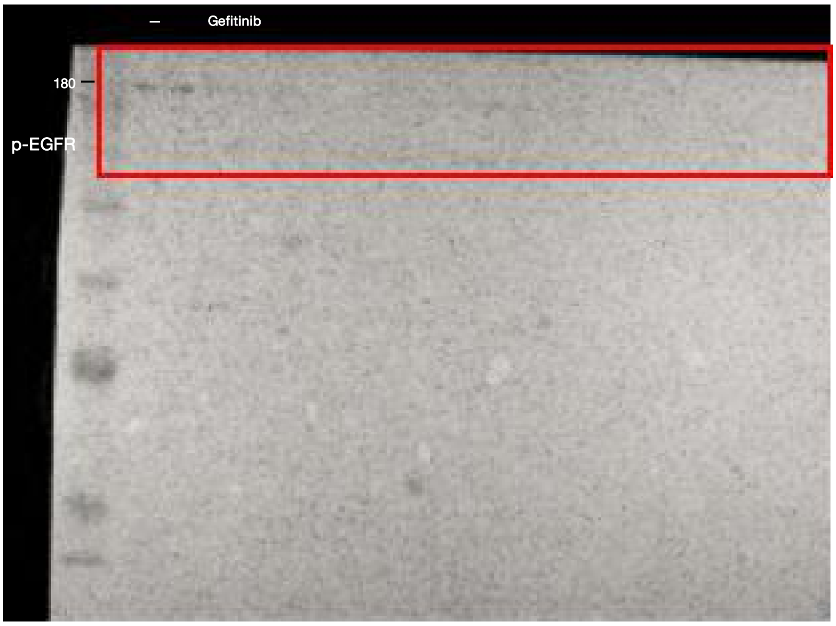

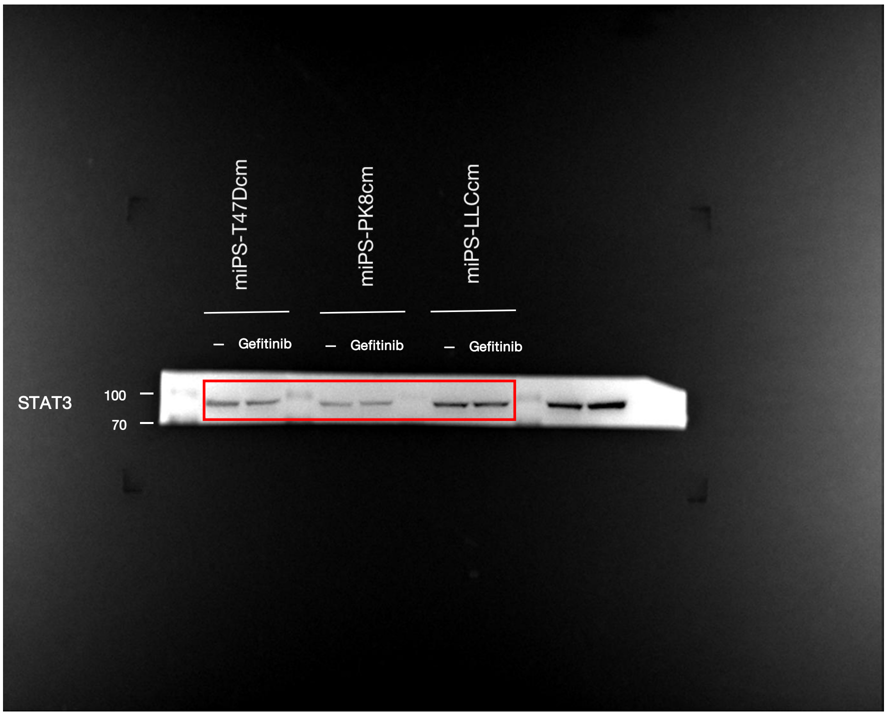

miPS-T47Dcm
miPS-PK8cm
miPS-LLCcm
– Gefitinib
– Gefitinib
– Gefitinib
STAT3
100
70

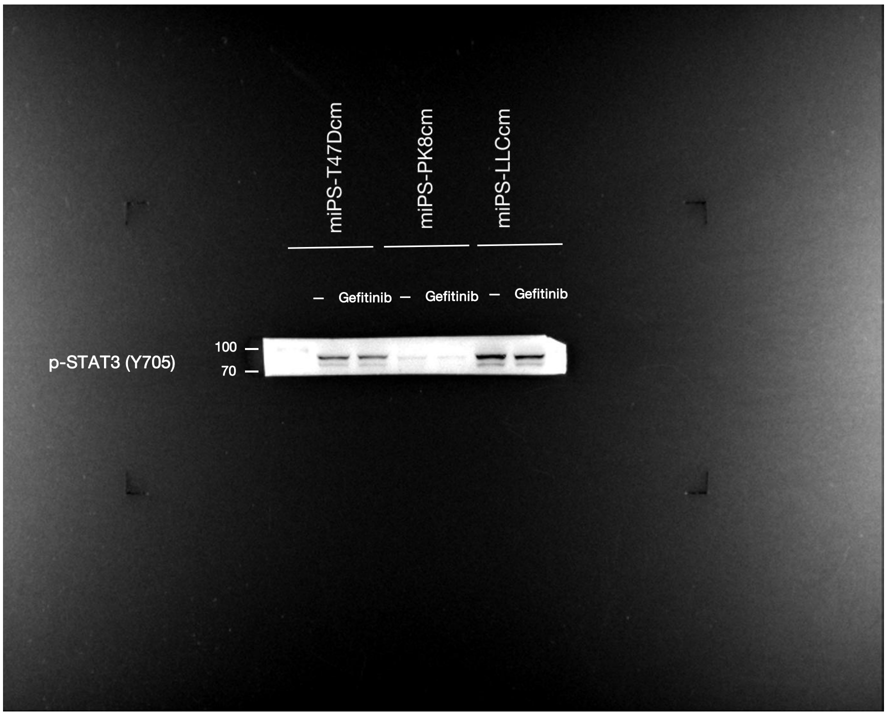

miPS-T47Dcm
miPS-PK8cm
miPS-LLCcm
– Gefitinib
– Gefitinib
– Gefitinib
p-STAT3 (Y705)
100
70

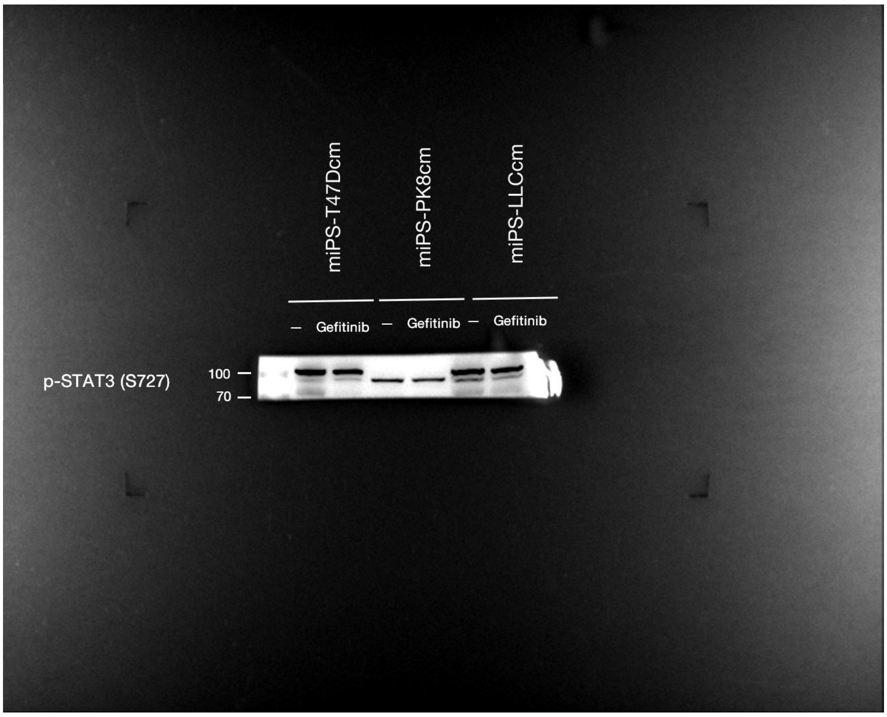

miPS-T47Dcm

in figure 2b

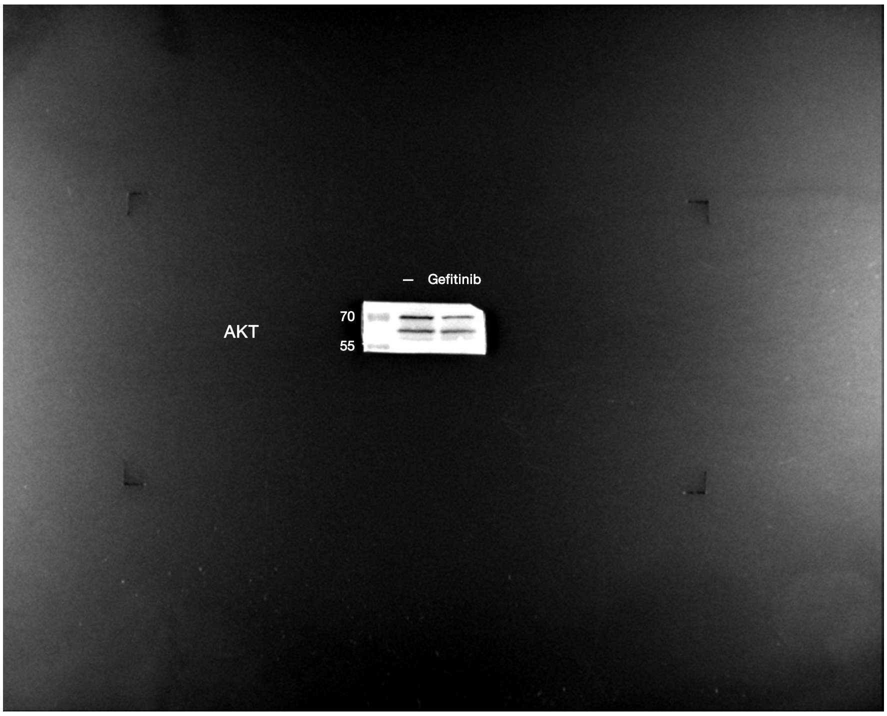

miPS-PK8cm

in figure 2b

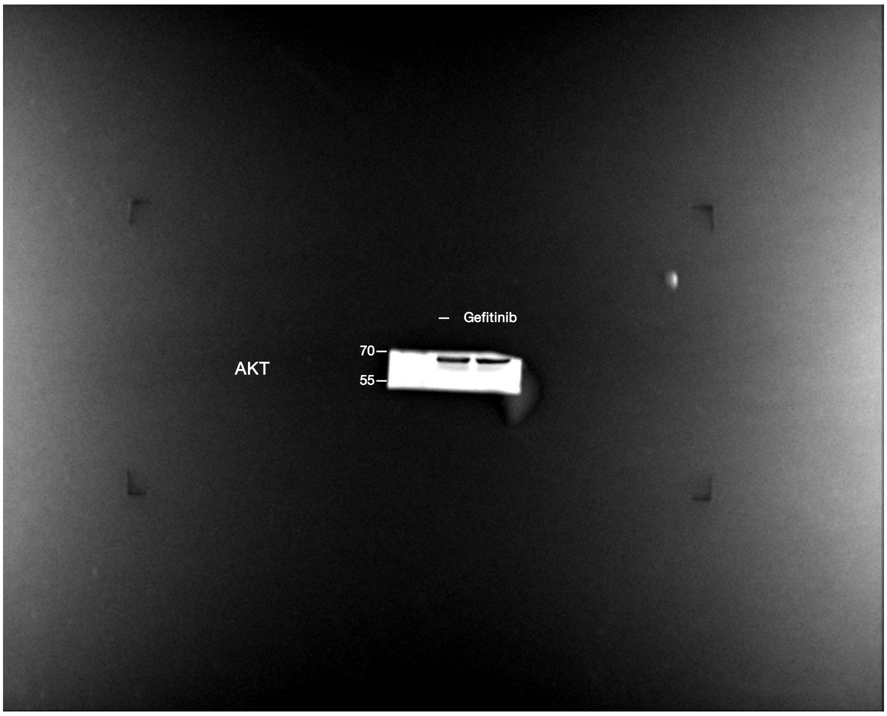

miPS-LLCcm

in figure 2b

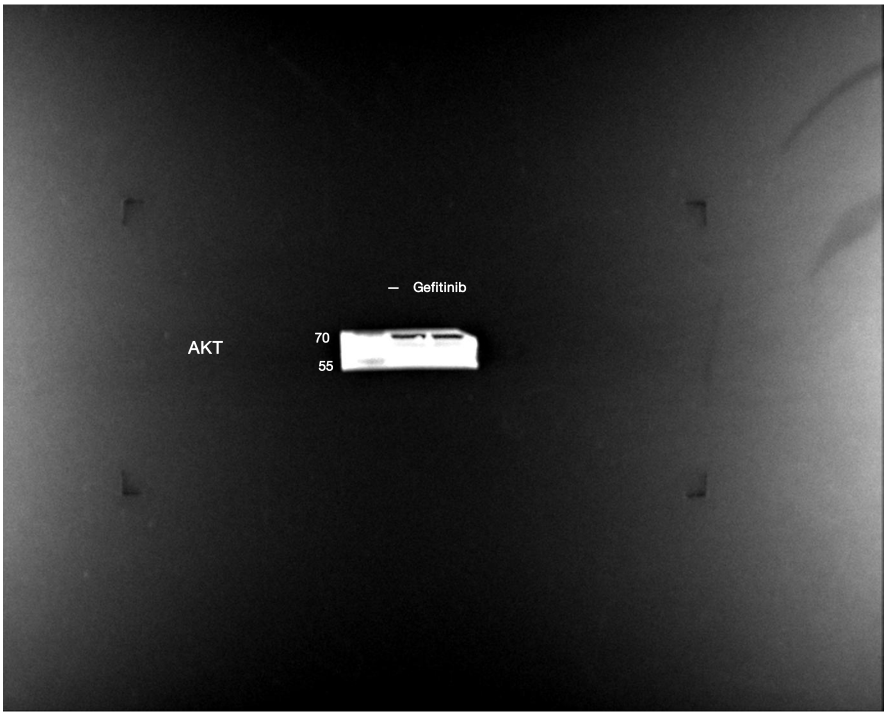

miPS-T47Dcm

in figure 2b

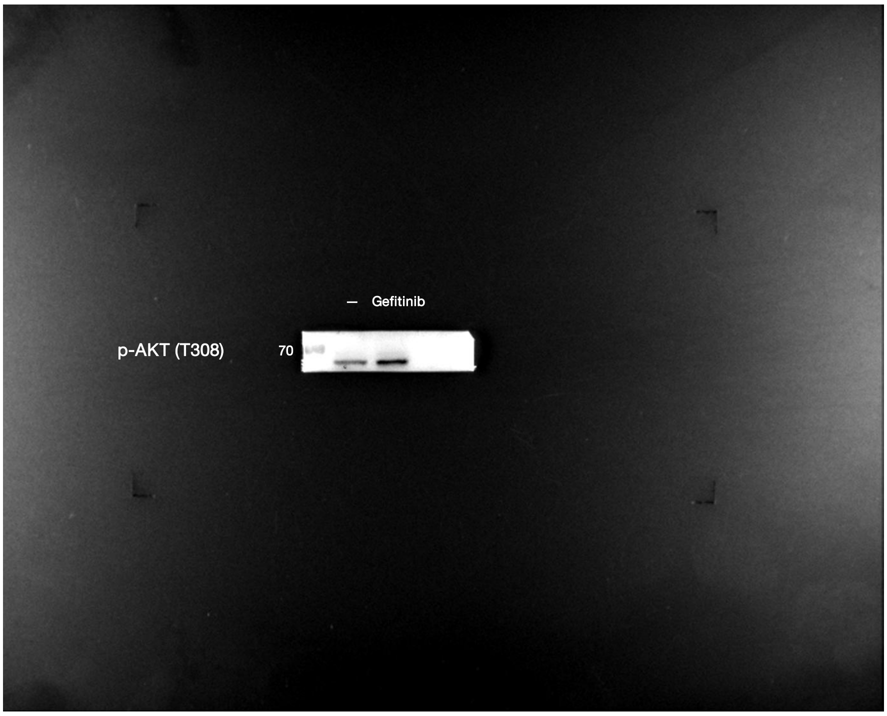

miPS-PK8cm

in figure 2b

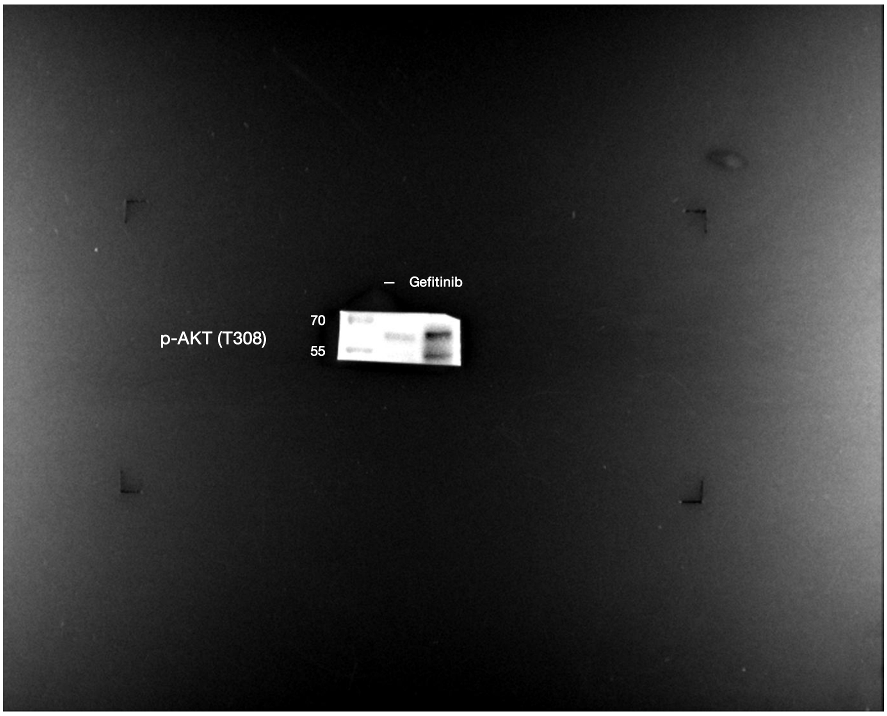

miPS-LLCcm

in figure 2b

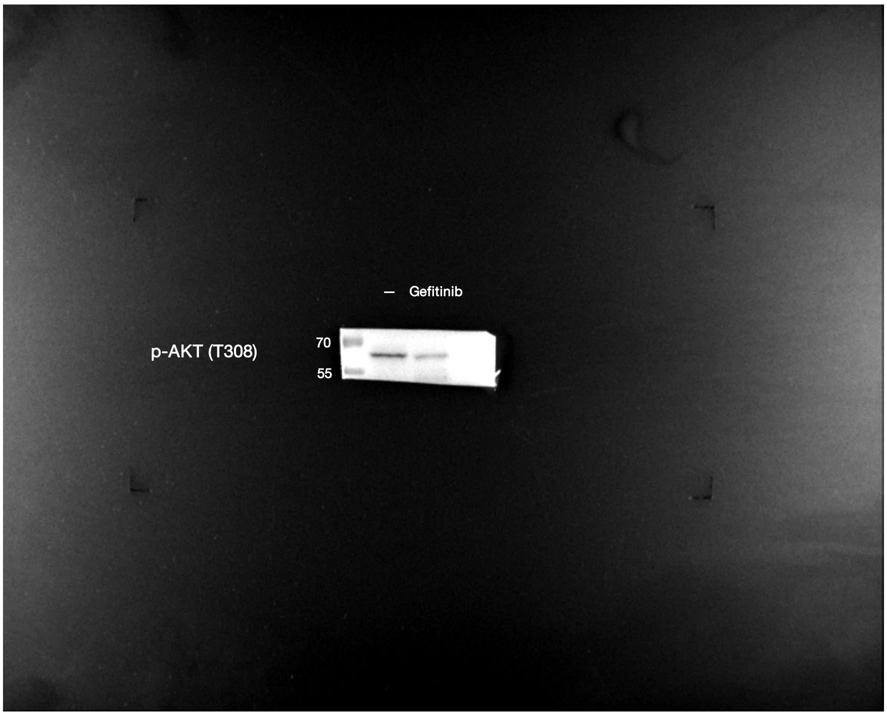

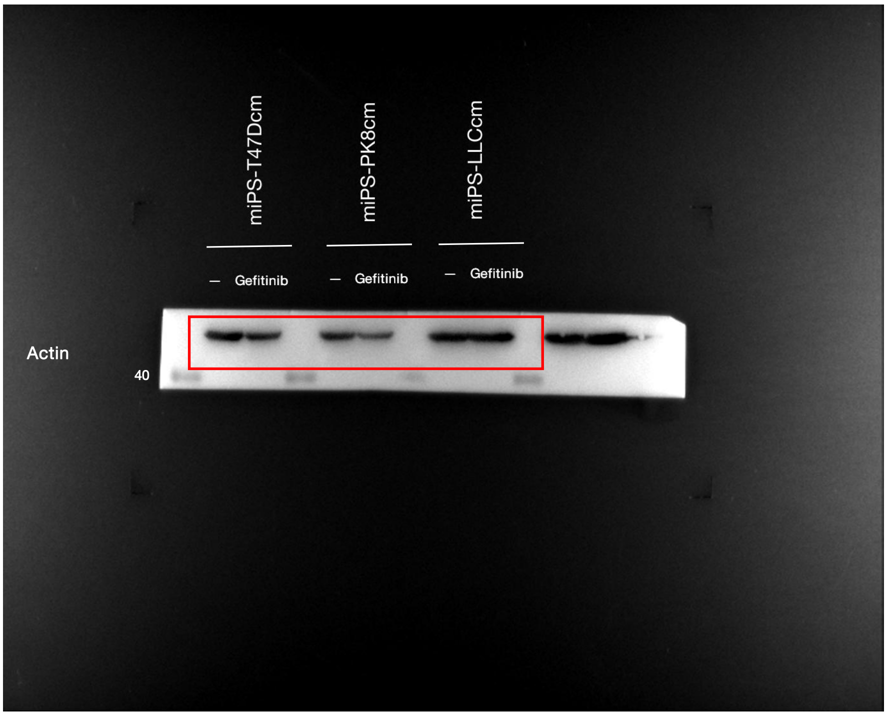

Supplementary Figure S4: Uncropped and unmodified original western blot figures related to Figure 2**b**.

miPS-T47Dcm

in figure 2c

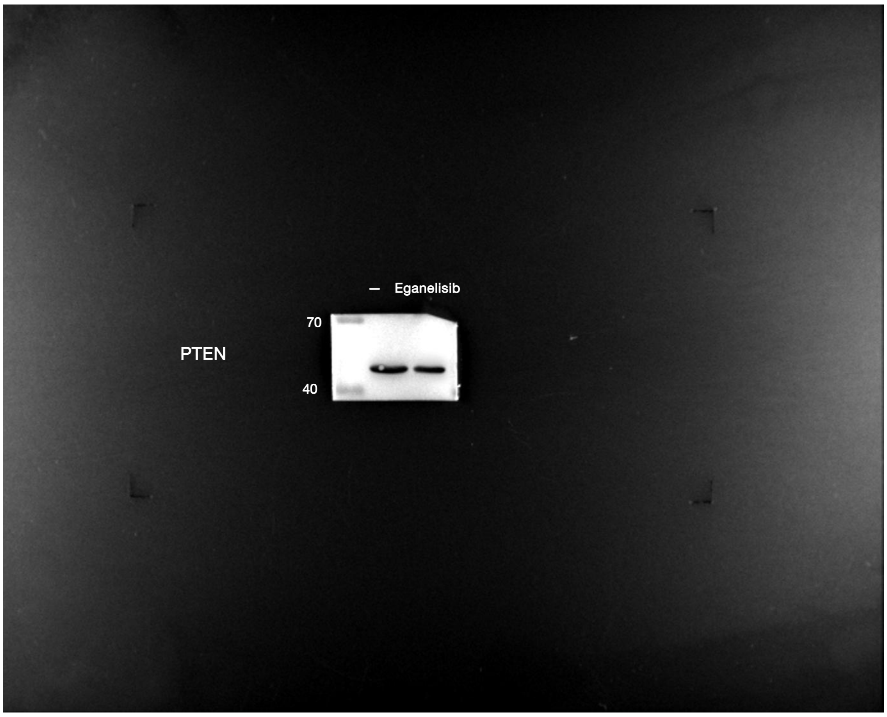

miPS-PK8cm

in figure 2c

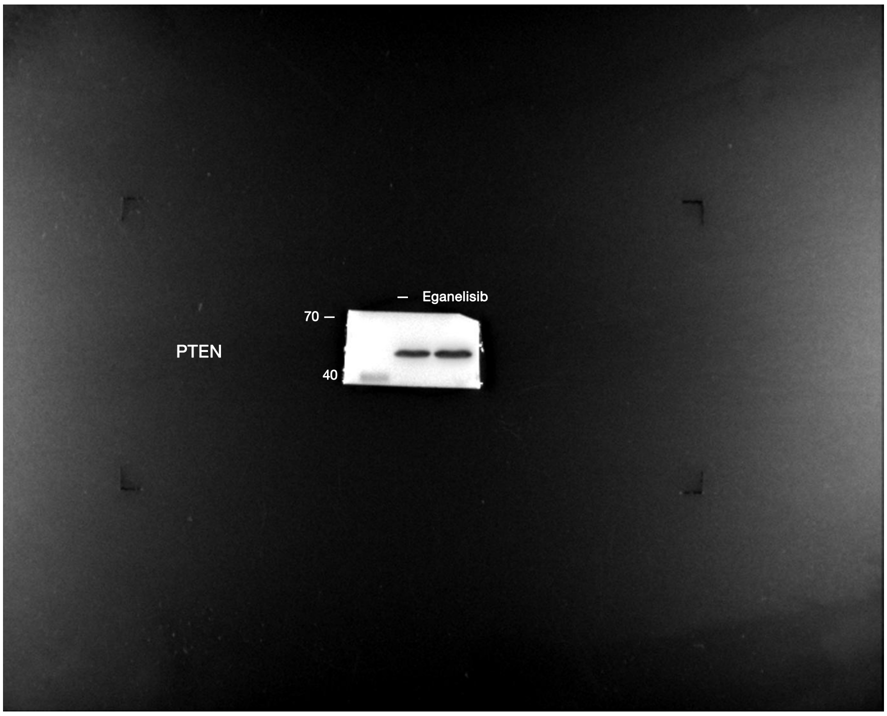

miPS-LLCcm

in figure 2c

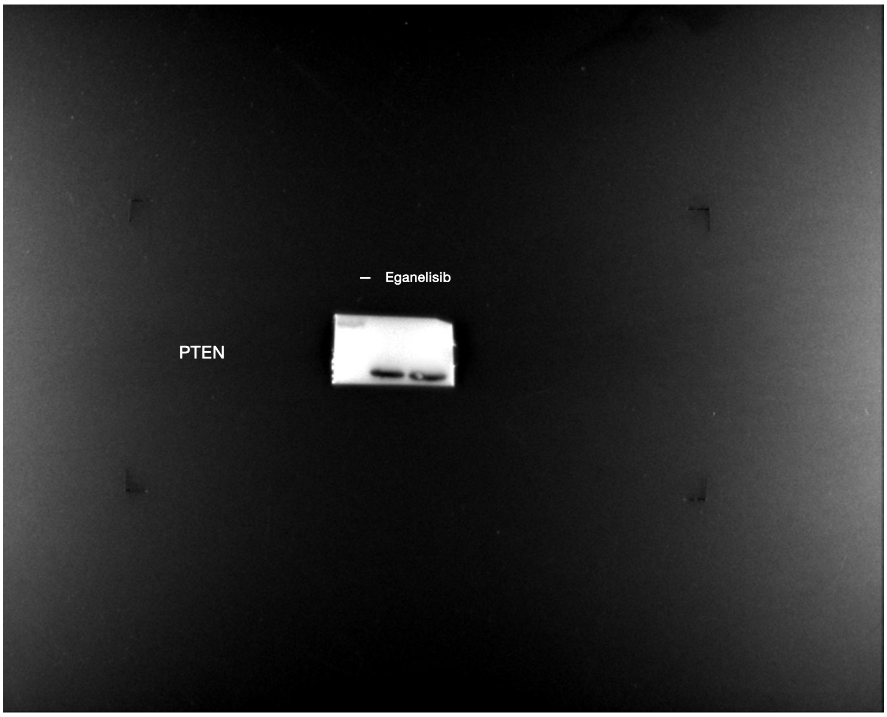

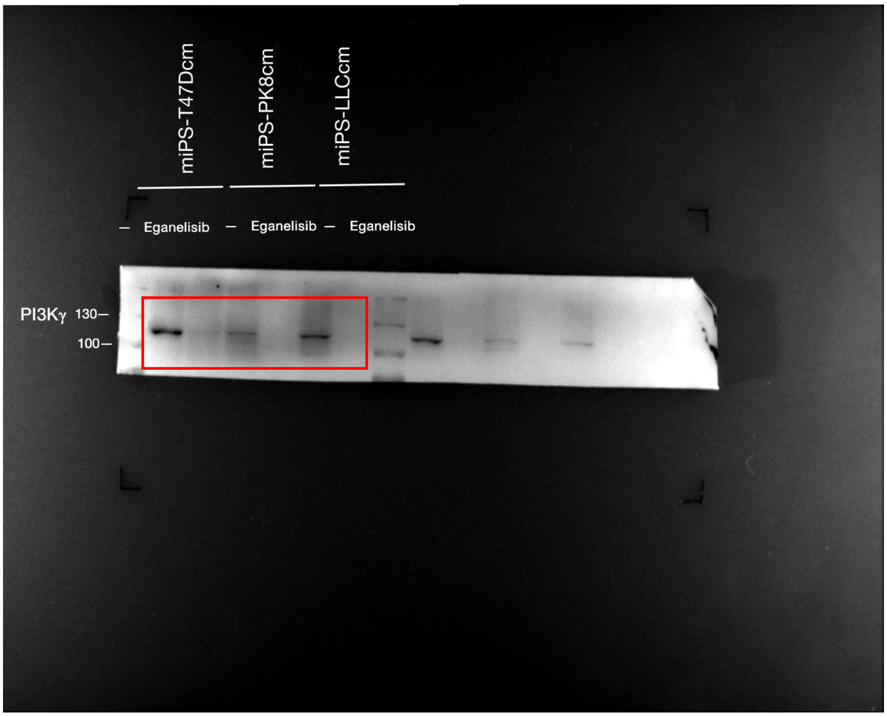

miPS-T47Dcm

in figure 2c

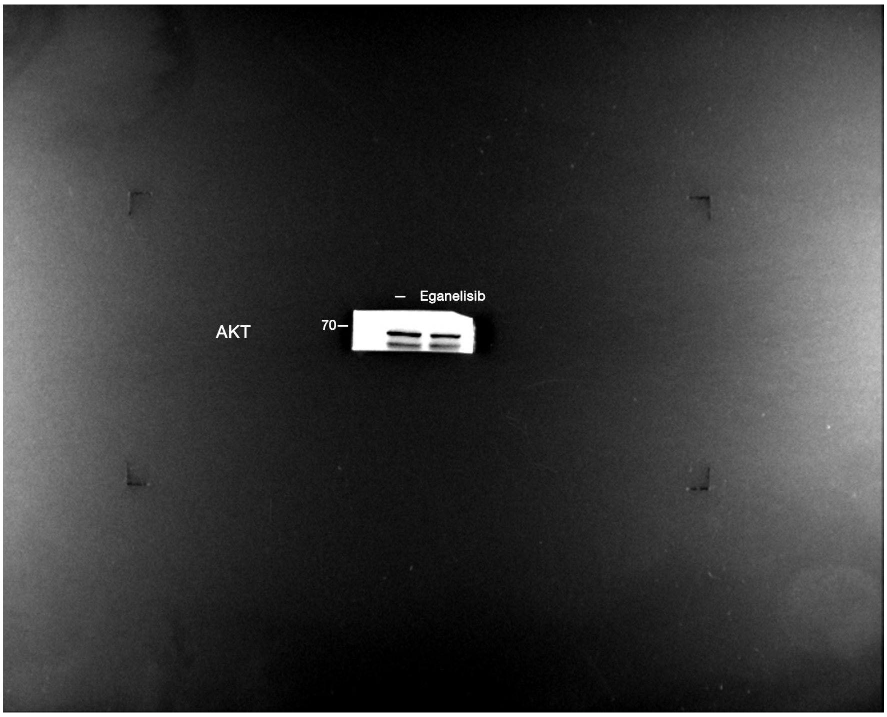

miPS-PK8cm

in figure 2c

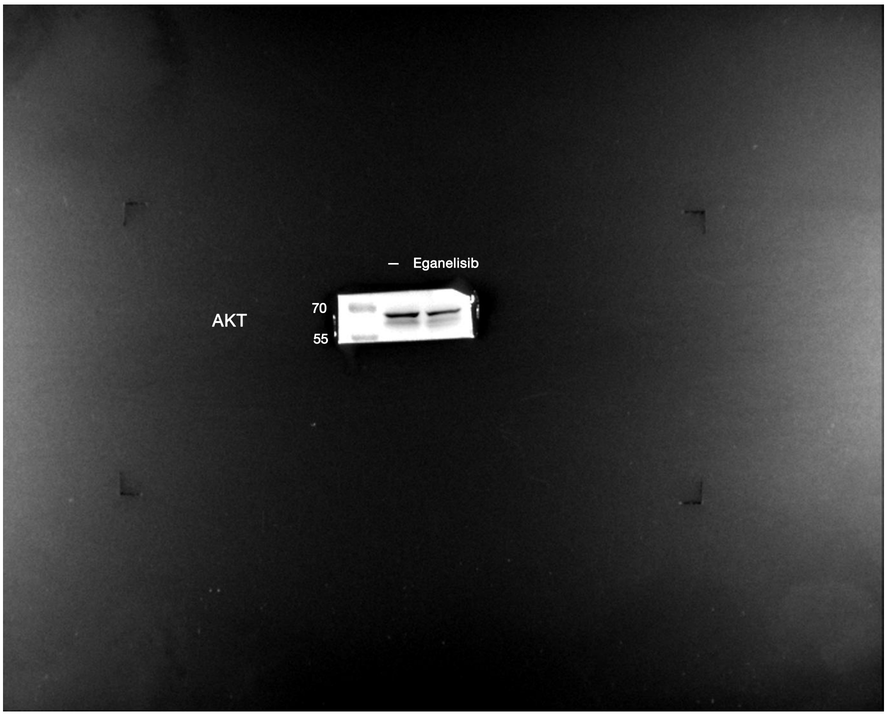

miPS-LLCcm

in figure 2c

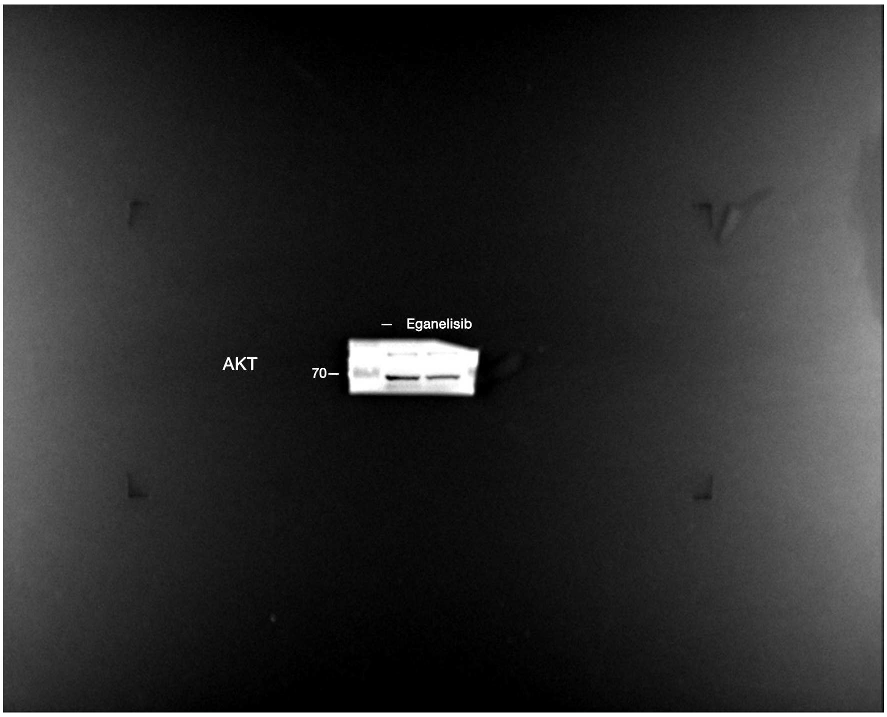

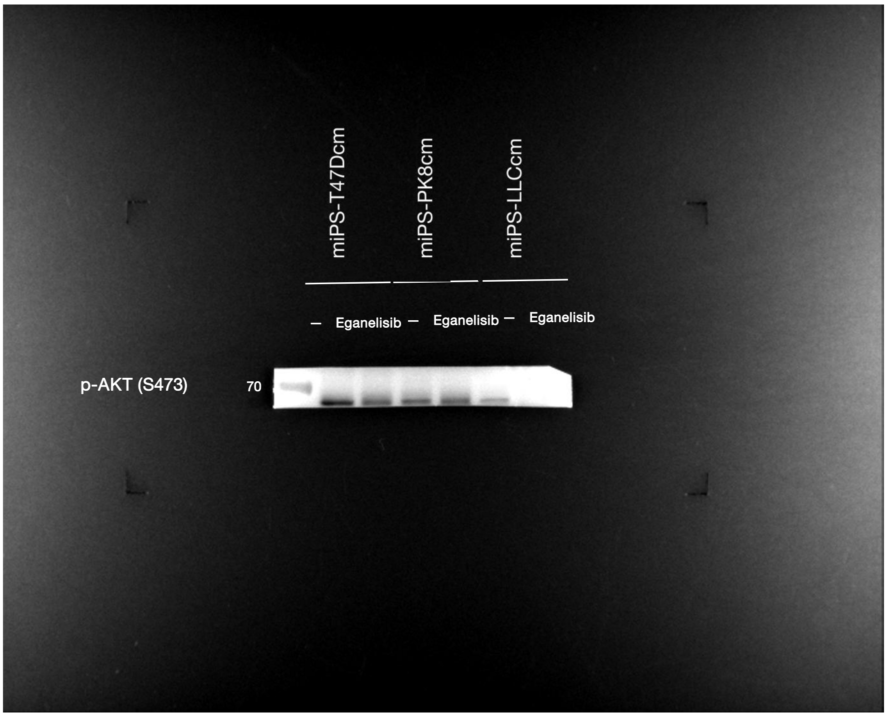

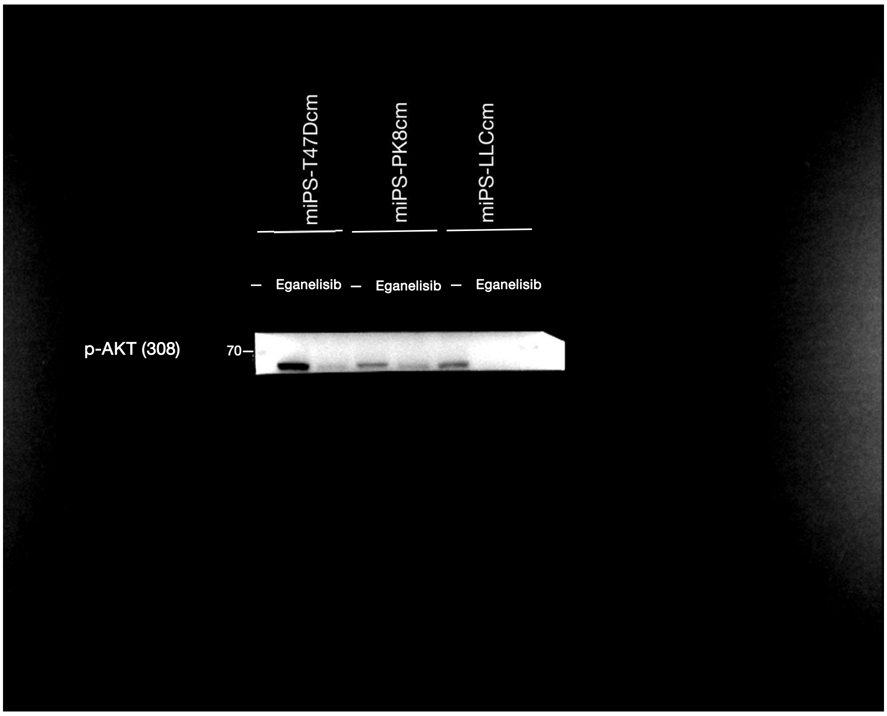
miPS-T47Dcm
miPS-PK8cm
miPS-LLCcm
– Eganelisib – Eganelisib – Eganelisib
p-AKT (308)
70

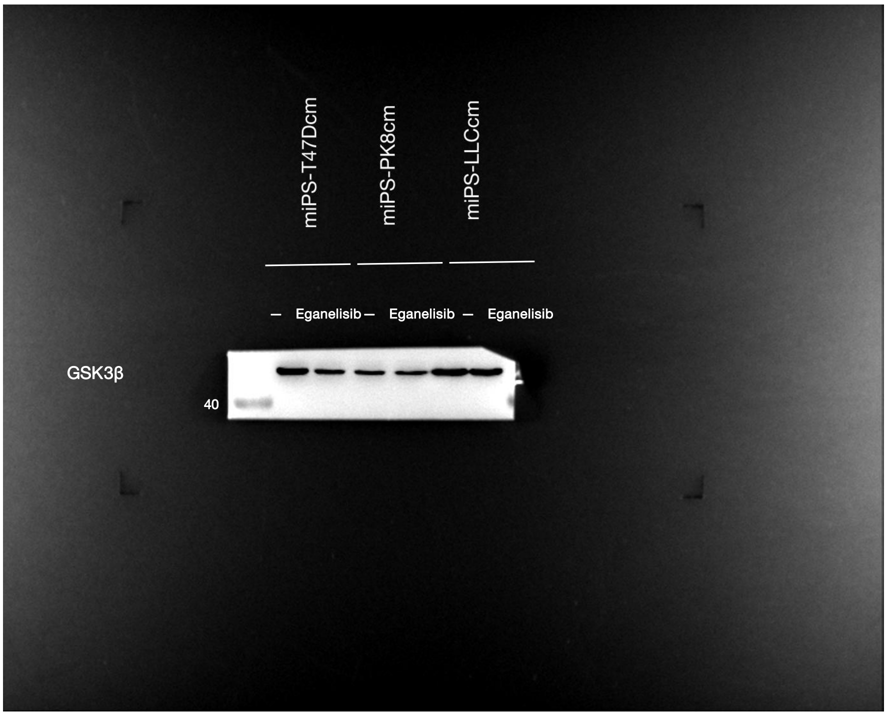
miPS-T47Dcm
miPS-PK8cm
miPS-LLCcm
– Eganelisib – Eganelisib – Eganelisib
GSK3β
40

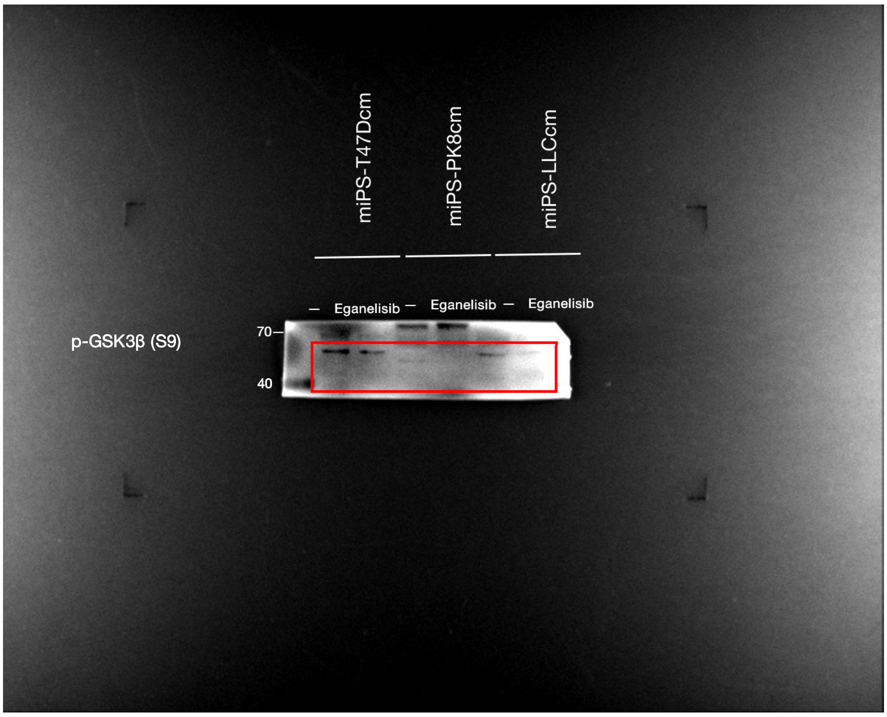

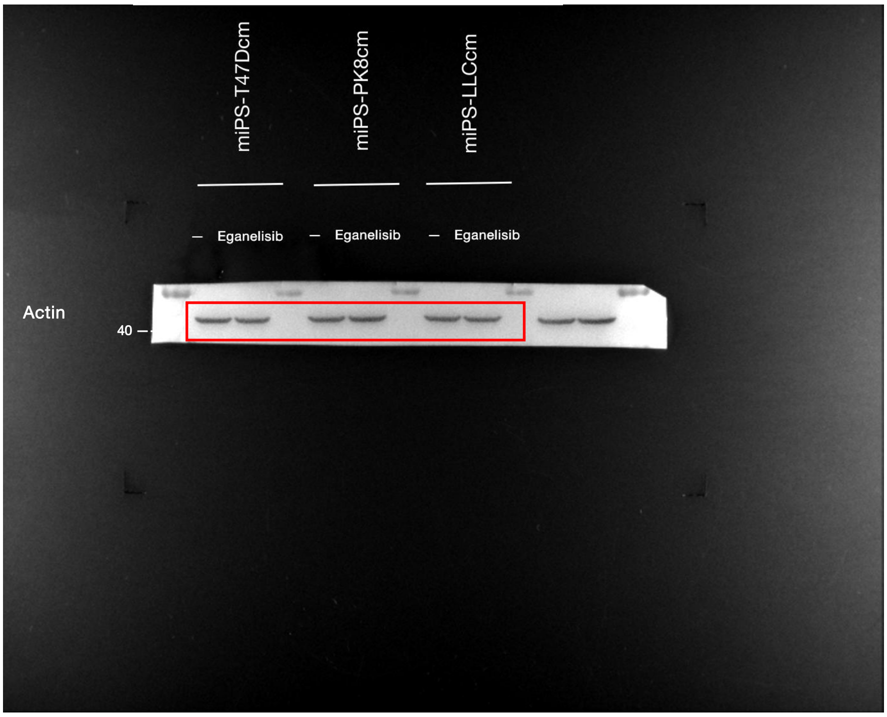

Supplementary Figure S5: Uncropped and unmodified original western blot figures related to Figure 2c.

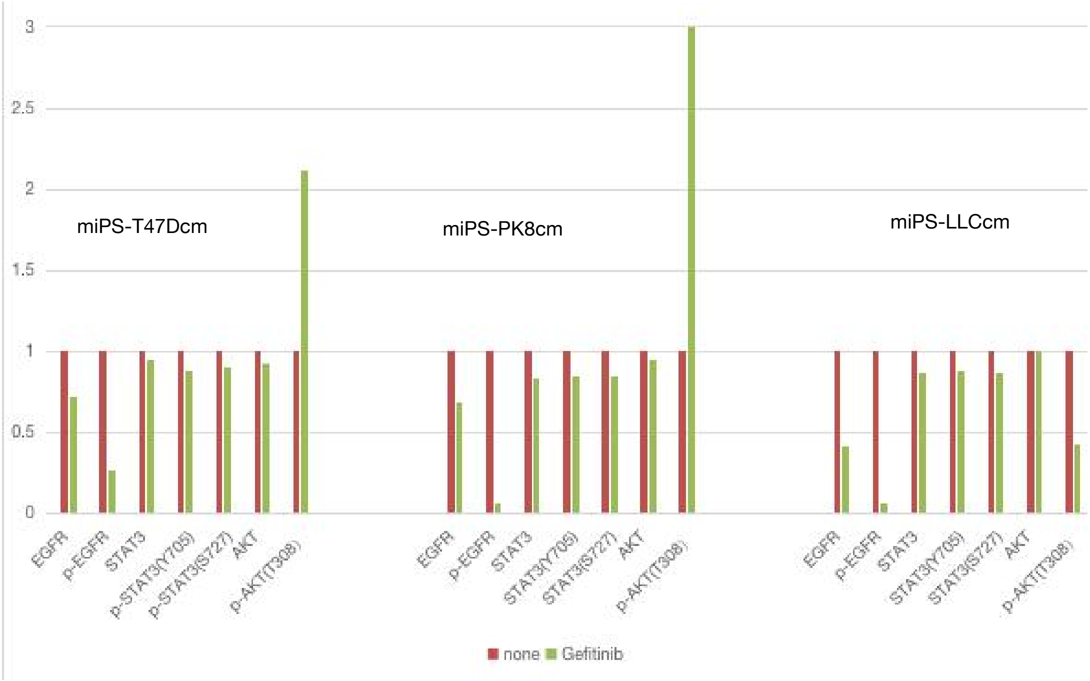

Supplementary Figure S6: Quantify the band density in 2**b**.

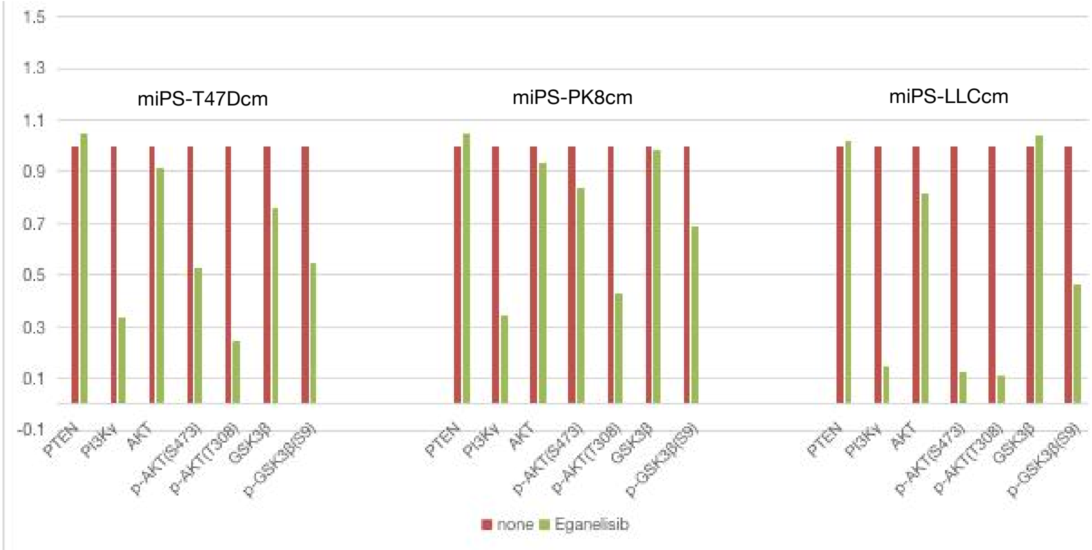

Supplementary Figure S7: Quantify the band density in 2**c**.
